# Multimodal CT radiomics-clinical ensemble machine learning model effectively predicts futile recanalization after endovascular treatment of acute ischemic stroke

**DOI:** 10.3389/fnins.2026.1838675

**Published:** 2026-05-12

**Authors:** Zhenxiong Wang, Yidong Gao, Pan Xu, Di Wu, Wuying Li, Huameng Huang, Weihua Deng, Honggang Xu, Xinhua Wei, Xing Li

**Affiliations:** 1 Department of Radiology, Guangzhou First People's Hospital, The Second Affiliated Hospital, School of Medicine, South China University of Technology, Guangzhou, China; 2 Department of Neurology, Guangzhou First People's Hospital, The Second Affiliated Hospital, School of Medicine, South China University of Technology, Guangzhou, China; 3 Department of Radiology, The Fifth Affiliated Hospital of Sun Yat-sen University, Zhuhai, China

**Keywords:** CT radiomics, futile recanalization, machine learning, predictive model, stroke

## Abstract

**Backgrounds:**

Futile recanalization (FR) poses a significant challenge in endovascular treatment and there is a lack of reliable predictive models for assessing treatment outcomes in stroke. The aim of this study is to develop a robust CT radiomics-clinical ensemble model that predicts FR in patients with acute ischemic stroke (AIS) following endovascular treatment (EVT) utilizing machine learning techniques.

**Methods:**

This study enrolled 101 patients diagnosed with AIS who underwent successful EVT. A total of 946 radiomics features were, respectively, extracted from non-contrast CT (NCCT), contrast-enhanced CT (CECT), and various CT perfusion maps (CBF, CBV, MTT, and TTP) using PyRadiomics prior to the endovascular intervention. Demographic characteristics, along with baseline clinical, laboratory, and angiographic variables, were incorporated as clinical features in the model analysis. Feature engineering was performed using SelectKBest. Five traditional machine learning algorithms were employed for modeling. The dataset was randomly split into a training cohort (*n* = 71, 70%) and an internal validation cohort (*n* = 30, 30%). Receiver operating characteristic (ROC) curves were utilized to evaluate the performance of each model.

**Results:**

Among the 101 patients, FR occurred in 66 individuals (65%), as determined by the modified Rankin Scale (mRS) at 90 days. The ensemble model integrating clinical data, NCCT, and CBV achieved the highest performance, with an area under the curve (AUC) of 0.918 using the CatBoost algorithm.

**Conclusion:**

The multimodal CT radiomics-clinical ensemble machine learning model demonstrated excellent predictive capability for identifying FR in AIS patients with large vessel occlusion prior to EVT.

## 1 Introduction

Endovascular therapy (EVT) has been endorsed by international guidelines for treating acute ischemic stroke (AIS) patients with large vessel occlusion (LVO) (Lin et al., 2019; Powers et al., 2019). The global implementation of these guidelines has led to improved clinical outcomes for stroke patients in recent years. Despite achieving successful recanalization (modified Thrombolysis in Cerebral Infarction [mTICI] 2b-3) in approximately 90% of cases with EVT, nearly half of the patients still experience an unfavorable functional outcome at 90 days (modified Rankin Scale [mRS] 3–6) (Goyal et al., 2016; Happi Ngankou et al., 2021; Jia et al., 2021). This situation is often referred to as futile recanalization (FR) or clinically ineffective reperfusion (CIR) (Hussein et al., 2010; Nie et al., 2023). FR presents significant challenges and confusion in the clinical management of AIS.

To prevent FR and identify risk factors prior to endovascular treatment, numerous potential predictors and predictive models have been proposed, showing promising potential (Brugnara et al., 2020; D’Anna et al., 2024; Da Ros et al., 2024; Su et al., 2021). However, many previous studies have relied on limited imaging or clinical data and traditional statistical methods, which hinder their ability to integrate multimodal parameters to explore the complex causes and mechanisms of FR and accurately predict outcomes. Therefore, it is essential to develop comprehensive methods for conducting a thorough analysis of various factors using diverse data modalities.

Radiomics is an innovative analytical technique that extracts extensive information regarding the characteristics of biological tissues from large imaging datasets, providing quantitative, high-dimensional, and mineable features for further analysis (Varghese et al., 2019). It has demonstrated exceptional performance in areas such as disease diagnosis and prognosis prediction, and shows considerable promise in stroke management (Chen et al., 2021; Luo et al., 2023). Machine learning, a core component of artificial intelligence, encompasses a set of computational methods that proficiently process complex patterns and nonlinear relationships within substantial datasets—capabilities that traditional statistical models struggle to achieve (Choy et al., 2018). Recent studies suggest that machine learning methods hold potential for predicting outcomes in AIS patients undergoing EVT; however, there is a lack of research applying radiomics and machine learning techniques specifically to predict FR (D’Anna et al., 2024; Da Ros et al., 2024).

This study aims to establish a preoperative ensemble model that effectively predicts FR using machine learning methods, incorporating multimodal radiomics features along with clinical, laboratory, and angiographic data. This approach seeks to enhance personalized management of stroke patients and ultimately identify those who would benefit most from endovascular treatment in clinical practice.

## 2 Materials and methods

**Figure 1:**
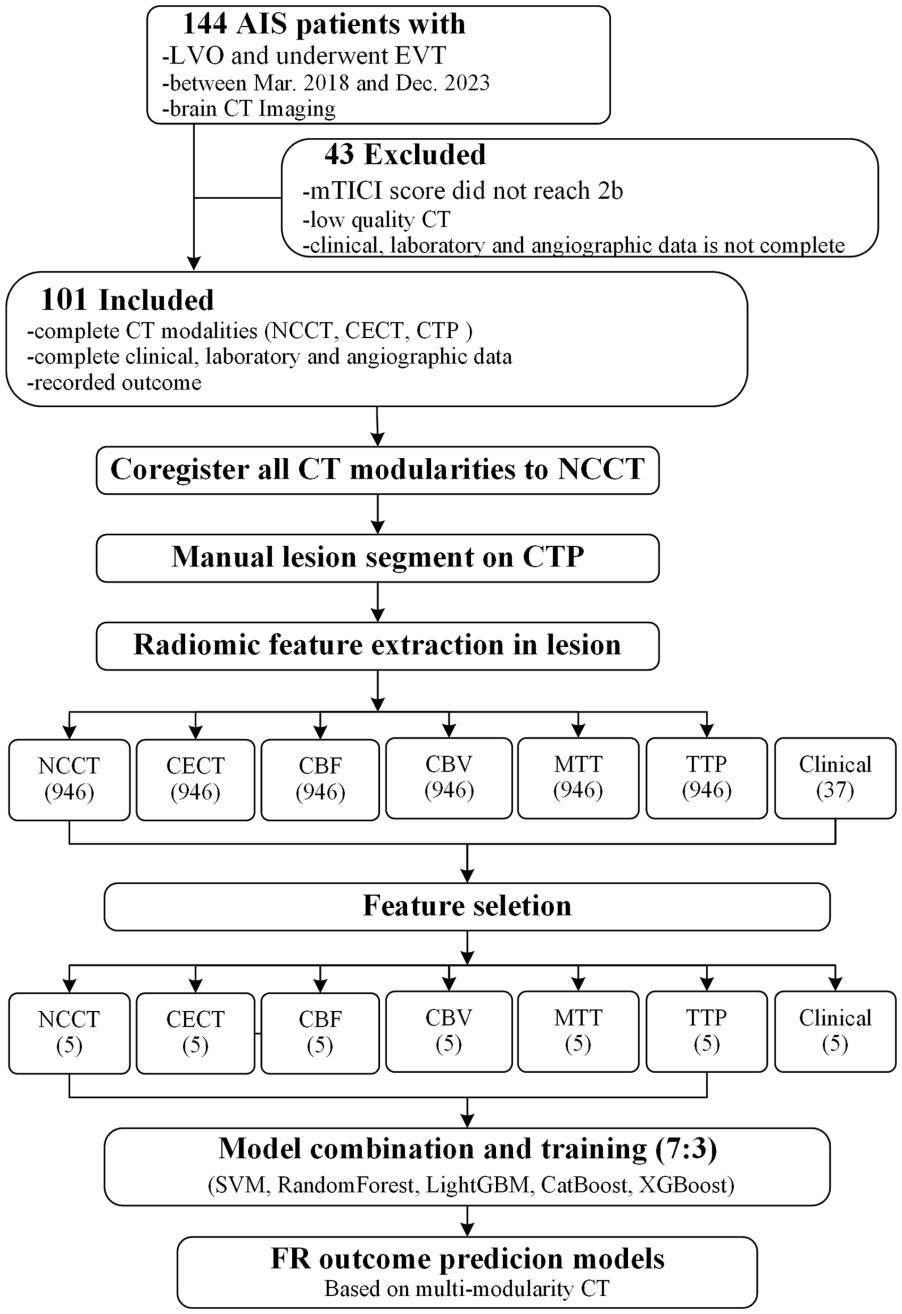
Patient selection process and study workflow. AIS, acute ischemic stroke; LVO, large vessel occlusion; EVT, endovascular therapy; mTICI, modified thrombolysis in cerebral infarction; NCCT, non-contrast brain CT; CECT, contrast-enhanced CT; CTP, CT perfusion; CBF, cerebral blood flow; CBV, cerebral blood volume; MTT, mean transit time; TTP, time-to-peak; SVM, Support vector machine.

**Figure 2:**
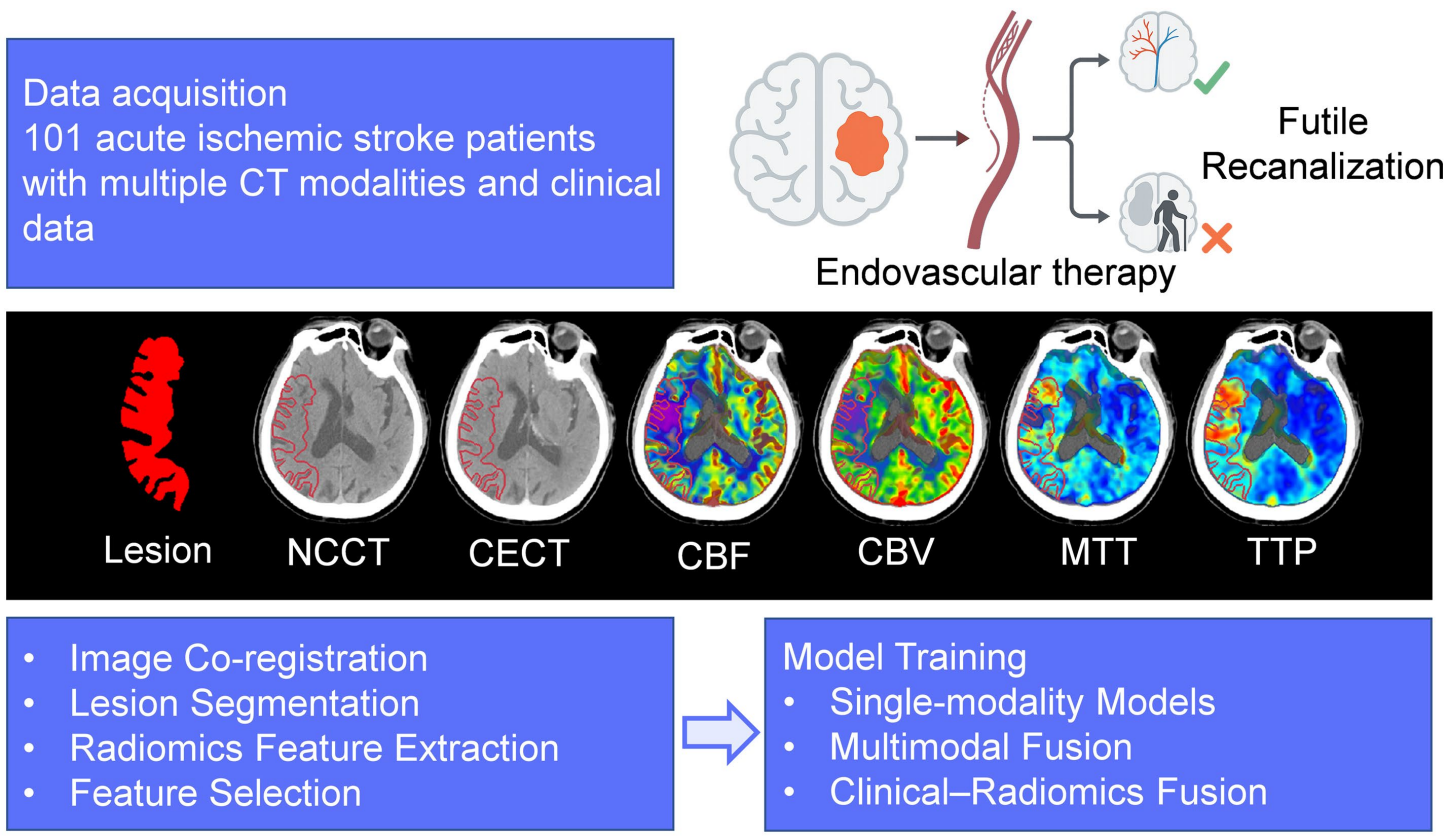
Overall study pipeline.

This retrospective study was approved by the institutional ethics committee. The analysis included a consecutive cohort of 144 patients diagnosed with acute ischemic stroke (AIS) due to large vessel occlusion (LVO) who underwent endovascular treatment (EVT) between March 2018 and December 2023. Inclusion criteria comprised clinically confirmed cases of AIS with LVO treated at our hospital. Multimodal CT imaging, including non-contrast CT (NCCT), contrast-enhanced CT (CECT), and multi-parametric CT perfusion (CTP), was performed prior to the endovascular intervention. Comprehensive clinical, laboratory, and angiographic data were collected. Exclusion criteria involved cases with motion artifacts and those where the mTICI score was below 2b. “Incomplete clinical data” referred to missing key variables required for modeling or outcome assessment, including unavailable baseline clinical/laboratory records, incomplete angiographic timing variables, or missing 90-day mRS data. “Poor image quality” referred to severe motion artifact, incomplete imaging coverage, or unsatisfactory inter-modality alignment on visual quality control that precluded reliable radiomics extraction. Ultimately, 101 patients were included in the subsequent research analysis after excluding 15 patients with unsuccessful recanalization and mTICI < 2b, 20 patients with incomplete clinical data, and 8 patients with poor image quality. The patient selection process and overall study pipeline are illustrated in Figures 1, 2.

### 2.1 Imaging protocols

All CT images were acquired on a 320-slice CT scanner (Aquilion ONE TSX-301A, Canon, Tokyo, Japan) with a standardized protocol. NCCT was performed first (matrix 512 × 512; typical 120 kVp; automatic mA modulation; slice thickness 1 mm). When clinically warranted, CECT was performed to evaluate vascular anatomy. Dynamic CTP was executed using a bolus-tracking protocol to create quantitative parametric maps: cerebral blood flow (CBF), cerebral blood volume (CBV), mean transit time (MTT), and time-to-peak (TTP). Perfusion maps were generated on the vendor workstation through the deconvolution of tissue time–attenuation curves. For the present radiomics analysis, only patients with complete multimodal datasets available for feature extraction were included in the final cohort. DICOM images from all six modalities (NCCT, CECT, CBF, CBV, MTT, and TTP) were exported for further analysis, adhering to the ALARA principles for dose management.

### 2.2 Image preprocessing and registration

Image preprocessing was conducted to ensure spatial and intensity consistency across all modalities before radiomics extraction. First, each modality was linearly registered to the subject’s NCCT in native space using FSL FLIRT (6–12 DOF affine with trilinear interpolation) to achieve sub-voxel alignment (Jenkinson et al., 2002). For CTP images, a time–space refinement process was applied before generation of parametric maps to reduce minor motion between dynamic frames. All co-registered images then underwent visual quality control by two imaging analysts to confirm acceptable alignment between lesion-containing regions across modalities; cases with unsatisfactory registration were excluded from further analysis. Prior to radiomics extraction, intensity clipping was applied to reduce the influence of outlier voxels, and z-score normalization was performed within the brain mask to improve cross-modality intensity comparability (Li et al., 2022; Pisani et al., 2025). Subsequently, during feature extraction, images were resampled to isotropic voxels and discretized according to the PyRadiomics/IBSI workflow described below.

### 2.3 Lesion segmentation

Two board-certified neuroradiologists, each with over 10 years of experience, independently delineated ischemic lesions using ITK-SNAP, referencing all modalities concurrently, including NCCT and perfusion maps. A consensus was reached for the final lesion mask for each case.

### 2.4 Radiomics feature extraction

**Figure 3:**
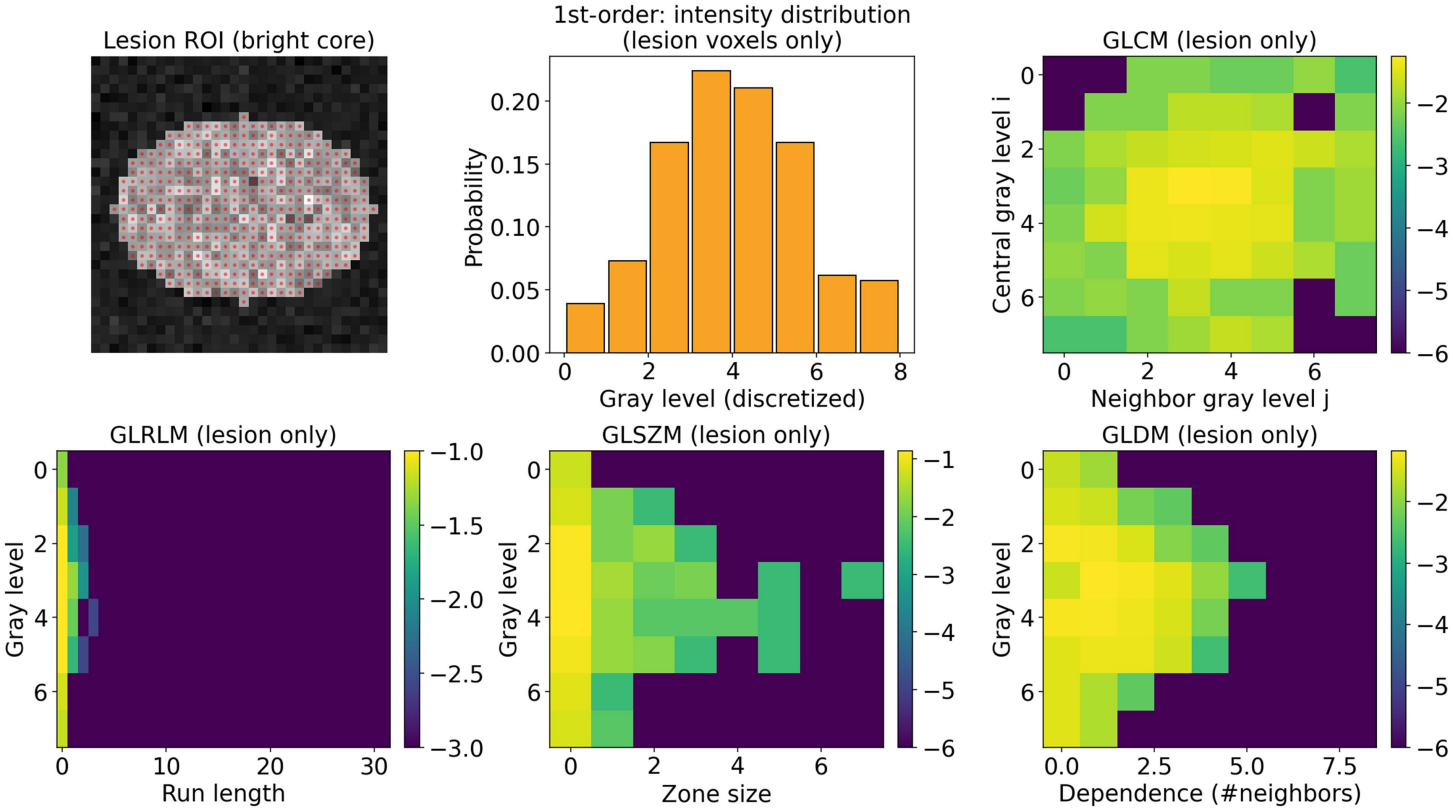
Illustration of radiomic feature composition. A single bright lesion region-of-interest (ROI) is shown together with the corresponding first-order intensity histogram and four classes of texture matrices—gray-level co-occurrence matrix (GLCM), gray-level run-length matrix (GLRLM), gray-level size-zone matrix (GLSZM), and gray-level dependence matrix (GLDM)—all computed within the lesion mask.

Radiomics features were extracted using PyRadiomics (v3.0), following the guidelines established by the Image Biomarker Standardisation Initiative (IBSI) to ensure reproducibility and comparability (van Griethuysen et al., 2017). Before extraction, the volumes were resampled to create isotropic voxels, and the intensity values were discretized with a fixed bin width of 5 HU; gray-level normalization was applied to create 100 bins for weighted images, ensuring consistency in texture computation. For each imaging modality, we calculated first-order statistics, shape features, and second-order texture metrics, which included the gray-level co-occurrence matrix (GLCM), gray-level run-length matrix (GLRLM), gray-level size-zone matrix (GLSZM), and gray-level dependence matrix (GLDM) (Figure 3). Higher-order features were generated from filtered images using the Laplacian of Gaussian filter with *σ* = 5 mm, alongside 3D wavelet sub-bands. In total, across both the original and ten filtered images, we extracted 946 features per modality (comprising 18 first-order, 22 GLCM, 16 GLRLM, 16 GLSZM, and 14 GLDM features per image type), resulting in a thorough multi-scale, multi-contrast descriptor set (Ferretti and Corino, 2025; Rezaeijo et al., 2025).

### 2.5 Clinical characteristics

This study gathered demographic and baseline clinical characteristics through a neurologist’s assessment. Collected data included sex, age, and medical history, covering hypertension, diabetes, smoking, alcohol consumption, atrial fibrillation, prior strokes, and coronary heart disease. Additionally, we recorded the TOAST classification for the subtypes of acute ischemic stroke, the occlusion territory (anterior or posterior circulation), baseline mRS and National Institutes of Health Stroke Scale (NIHSS) scores, alongside Glasgow Coma Scale (GCS) scores and the Alberta Stroke Program Early CT Score (ASPECTS). Laboratory parameters included glycated hemoglobin (HbA1c), homocysteine (Hcy), low-density lipoprotein (LDL), high-density lipoprotein (HDL), total cholesterol, triglycerides, uric acid, creatinine, C-reactive protein (CRP), cystatin C (Cys-C), brain natriuretic peptide (BNP), D dimer, neutrophil, lymphocyte, the neutrophil/lymphocyte ratio (NLR), and fibrinogen. Interventional angiographic variables were documented by the treating neurointerventionalist, including the type of anesthesia (general or local), bridging therapy, number of thrombectomy passes, and the intervals from symptom onset to puncture and recanalization, as well as the extent of recanalization mTICI score. Univariate screening guided subsequent feature selection, and patient clinical outcomes were evaluated using the mRS score at 90 days post-intervention. FR was defined as an unfavorable functional outcome, indicated by an mRS score > 2 despite a technically successful intervention (mTICI ≥ 2b). We adopted this definition because mRS > 2 at 90 days after successful recanalization is commonly used in prior EVT studies to represent loss of functional independence despite angiographic success (Hussein et al., 2010; Nie et al., 2023).

### 2.6 Feature engineering and dimensionality reduction

We utilized univariate ANOVA F-tests (SelectKBest) to rank features based on their association with the binary outcome within the training data only. To preserve the diversity and interpretability of modalities, we retained the top 5 radiomics features from each modality for the image-only models. Within each cross-validation fold, the retained features were then standardized (zero-mean, unit variance) using parameters estimated from the corresponding training subset only (Pedregosa et al., 2011).

### 2.7 Model development and training

The dataset was stratified based on outcomes and randomly divided into two partitions: 70% for training and 30% for testing. We trained five classifiers: Support Vector Machine (SVM) with RBF kernel, Random Forest, Light Gradient Boosting Machine (LightGBM), Extreme Gradient Boosting (XGBoost), and Categorical Boosting (CatBoost) (Chen and Guestrin, 2016; Ke et al., 2017; Ostroumova et al., 2017; Tibshirani, 2018). For multimodal fusion models, concatenated feature sets subsequently underwent recursive feature elimination (RFE) within the same training subset before classifier fitting. To address class imbalance, RandomOverSampler/SMOTE from imbalanced-learn was then applied only to the training portion of each fold, thereby avoiding data leakage (Lemaître et al., 2017). Hyperparameters, including learning rate, maximum depth, L1/L2 regularization, and number of estimators, were optimized by grid search within the 5-fold cross-validation framework using the 70% training partition only. After model selection, the best-performing configuration was refit on the complete training partition, and the untouched 30% test set was used once for final performance evaluation (Li et al., 2022).

### 2.8 Ensemble multimodal strategy

**Figure 4:**
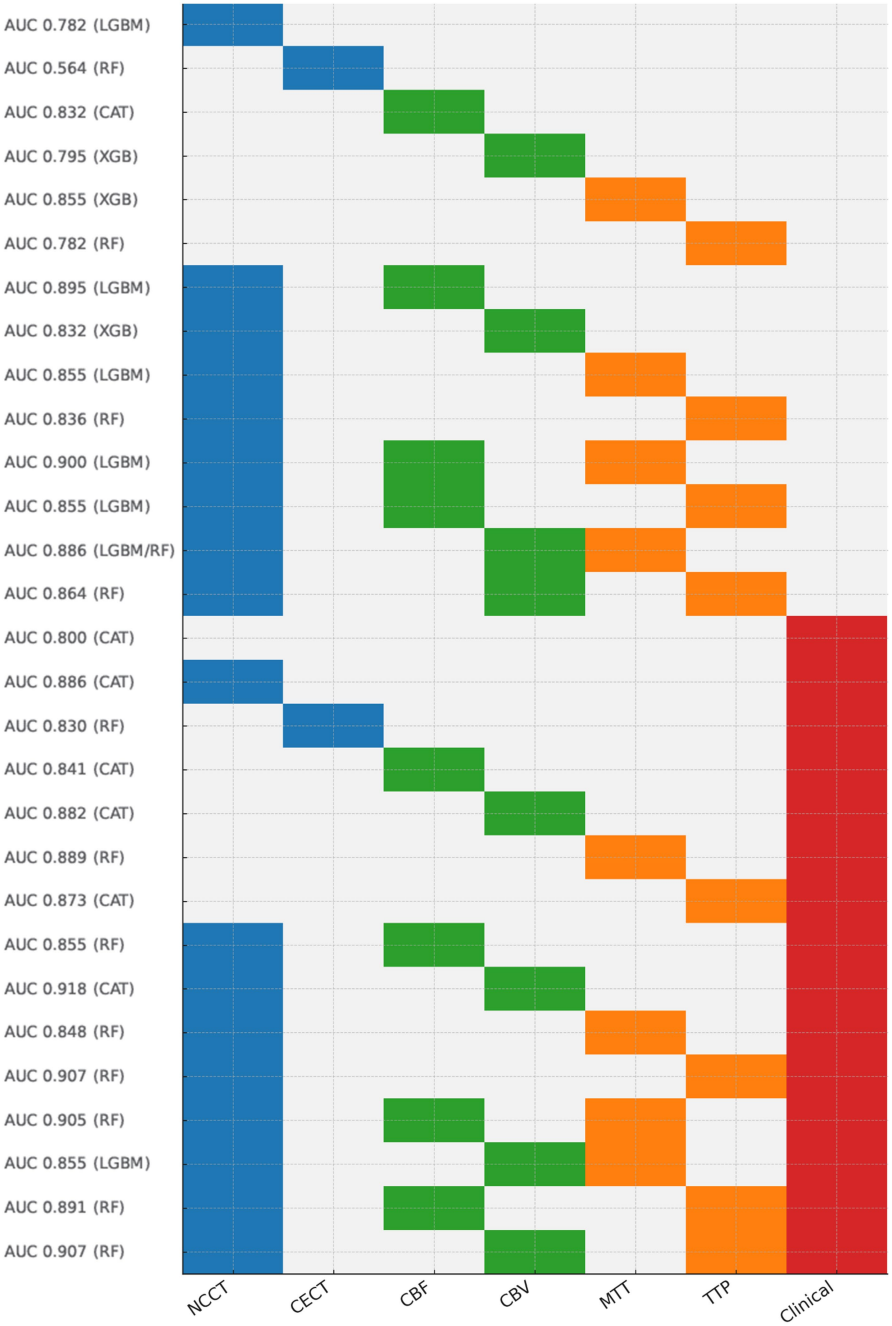
Top AUCs of all models and their fusion strategies. AUC, the area under the ROC curve; LGBM, Light Gradient Boosting Machine; RF, Random Forest; CAT, Categorical Boosting; XGB, Extreme Gradient Boosting.

We developed (i) single-modality CT radiomics models for each of the six modalities; (ii) dual-modality models by concatenating feature vectors from NCCT with one CTP map (e.g., NC + CBF, NC + CBV, NC + MTT, NC + TTP); and (iii) tri-modality models (e.g., NC + CBV + TTP). For clinical-imaging fusion, we selected five preoperative clinical variables (Cys-C, lymphocyte, NIHSS, onset-to-recanalization time, ASPECTS) using the same selection procedure and combined them with the radiomics features for joint modeling (Figure 4). We opted for early fusion (feature-level concatenation) to enhance transparency and ensured consistent preprocessing across all pipelines. To mitigate dimensionality inflation in higher-order fusions, we limited the selection to the top features from each modality and re-applied RFE within each fusion scenario (Wu et al., 2025).

### 2.9 Performance evaluation

Primary discrimination was assessed by the area under the receiver operating characteristic (ROC) curve (AUC), with results compared using DeLong’s test (DeLong et al., 1988). Additionally, we reported metrics such as accuracy, precision (positive predictive value), recall (sensitivity), specificity, and F1-score at the Youden-optimized threshold. Model calibration was assessed using calibration curves and Brier scores, while decision curve analysis quantified the net benefit across clinically relevant thresholds (Vickers and Elkin, 2006).

## 3 Results

In terms of clinical outcomes (mRS) for AIS patients 90 days after EVT, FR was observed in 66/101 patients (65%), with 35 patients achieving meaningful recanalization. Figure 4 illustrates top AUCs of all models. The details of the multimodal dataset and model construction are outlined below. In summary, the multimodal CT radiomics-clinical ensemble model outperformed others, while both CT radiomics and clinical models demonstrated strong predictive capabilities for FR in AIS patients undergoing EVT. Notably, XGBoost and CatBoost emerged as the most consistently effective classifiers for the complex radiomics-clinical multimodal data among the five classifiers.

### 3.1 Demographic and clinical characteristics

Baseline clinical, laboratory, and angiographic characteristics of patients with FR compared to those with meaningful recanalization are summarized in Table 1. In summary, the FR group was older than the meaningful recanalization group (69.56 vs. 61.03; *p* = 0.002) and exhibited a higher incidence of atrial fibrillation (27.3% vs. 0%; *p* < 0.001). Additionally, the FR group had higher median baseline mRS (5 [4, 5] vs. 4 [3, 4]; *p* < 0.001) and NIHSS scores (13 [8, 18] vs. 8 [6, 10]; *p* < 0.001), alongside lower GCS (10 [6, 14] vs. 13 [11, 15]; *p* = 0.038) and ASPECTS (8 [7, 8] vs. 9 [8, 9]; *p* < 0.001). The FR group also had higher baseline serum Cys-C (1.11 vs. 0.90; *p* = 0.010) and BNP levels (1139.64 vs. 861.32; *p* = 0.026), a lower lymphocyte (1.38 vs. 1.72; *p* = 0.006), and higher NLR (10.00 vs. 4.98; *p* = 0.025). Furthermore, the FR group experienced longer onset-to-start of endovascular treatment times (429.09 vs. 374.11 min; *p* = 0.048) and longer onset-to-recanalization times (518.94 vs. 429.06 min; *p* = 0.002). The remaining characteristics did not show significant differences between the two groups.

**Table 1:** Baseline characteristics by outcome.

Variable	Meaningful recanalization *N* = 35^1^	Futile recanalization *N* = 66^1^	*p-*value^2^
Clinical			
Sex			0.087
Female	9 (26%)	29 (44%)	
Male	26 (74%)	37 (56%)	
Age (years)	61.03 ± 11.88	69.56 ± 13.88	**0.002**
Hypertension (yes)	68.6%	56.1%	0.286
Diabetes (yes)	25.7%	34.8%	0.379
Smoking (yes)	34.3%	24.2%	0.351
Drinking (yes)	8.6%	12.1%	0.743
Atrial fibrillation (yes)	0.0%	27.3%	**<0.001**
Prior stroke			>0.999
No	28 (80%)	52 (80%)	
Yes	7 (20%)	13 (20%)	
Unknown	0	1	
Coronary artery disease (yes)	5.7%	12.1%	0.487
TOAST classification			0.400
Large-artery atherosclerosis	26 (74%)	41 (62%)	
Cardioembolism	7 (20%)	21 (32%)	
Stroke of other determined etiology	2 (5.7%)	4 (6.1%)	
Occlusion territory			>0.999
Anterior circulation	29 (83%)	54 (82%)	
Posterior circulation	6 (17%)	12 (18%)	
mRS (baseline)	4 [3, 4]	5 [4, 5]	**<0.001**
NIHSS (baseline)	8 [6, 10] (3–20)	13 [8, 18] (1–31)	**<0.001**
Glasgow Coma Scale (GCS)	13 [11, 15] (3–15)	10 [6, 14] (3–15)	**0.038**
ASPECTS	9 [8, 9] (7–9)	8 [7, 8] (6–9)	**<0.001**
Laboratory			
Glycated hemoglobin (HbA1c)	6.22 ± 1.39	6.46 ± 1.84	0.828
Homocysteine (Hcy)	13.74 ± 9.43	14.33 ± 7.98	0.516
Low-density lipoprotein (LDL)	2.68 ± 0.73	3.00 ± 1.14	0.261
High-density lipoprotein (HDL)	0.98 ± 0.23	1.05 ± 0.27	0.237
Total cholesterol	4.27 ± 0.90	4.62 ± 1.36	0.418
Triglycerides	1.32 ± 0.57	1.43 ± 1.06	0.814
Uric acid	376.91 ± 109.18	391.74 ± 136.03	0.633
Creatinine	85.40 ± 51.75	88.20 ± 29.42	0.099
C-reactive protein (CRP)	11.90 ± 14.01	23.20 ± 36.14	0.128
Cystatin C	0.90 ± 0.51	1.11 ± 0.58	**0.010**
Brain natriuretic peptide (BNP)	861.32 ± 3,097.90	1,139.64 ± 1,870.95	**0.026**
D-dimer	2,020.20 ± 4,565.42	1,914.58 ± 2,686.48	0.237
Neutrophils	7.33 ± 2.14	8.38 ± 4.18	0.348
Lymphocytes	1.72 ± 0.71	1.38 ± 0.84	**0.006**
Neutrophil-to-lymphocyte ratio (NLR)	4.98 ± 2.72	10.00 ± 10.82	**0.025**
Fibrinogen	3.44 ± 1.28	3.58 ± 1.13	0.309
Angiographic			
Anesthesia type			>0.999
Local	30 (86%)	56 (85%)	
General	5 (14%)	10 (15%)	
Bridging therapy (Yes)	22.9%	16.7%	0.593
Onset-to-puncture, min	374.11 ± 105.51	429.09 ± 136.15	**0.048**
Onset-to-recanalization, min	429.06 ± 110.56	518.94 ± 136.68	**0.002**
Number of retrieval attempts			0.061
1	22 (63%)	29 (44%)	
2	12 (34%)	23 (35%)	
3	1 (2.9%)	12 (18%)	
4	0 (0%)	2 (3.0%)	
mTICI (post-recanalization)			0.141
2b	5 (14%)	19 (29%)	
3	30 (86%)	47 (71%)	

1 *n* (%), Mean ± SD.

2 Pearson’s Chi-squared test, Fisher’s exact test, Wilcoxon rank sum test. Bold values mean *p* < 0.05, and was considered statistically significant.

mRS, modified Rankin Scale; NIHSS, National Institutes of Health Stroke Scale; ASPECTS, Alberta Stroke Program Early CT Score; mTICI, modified Thrombolysis in Cerebral Infarction.

### 3.2 Single-modality CT radiomics models

**Figure 5:**
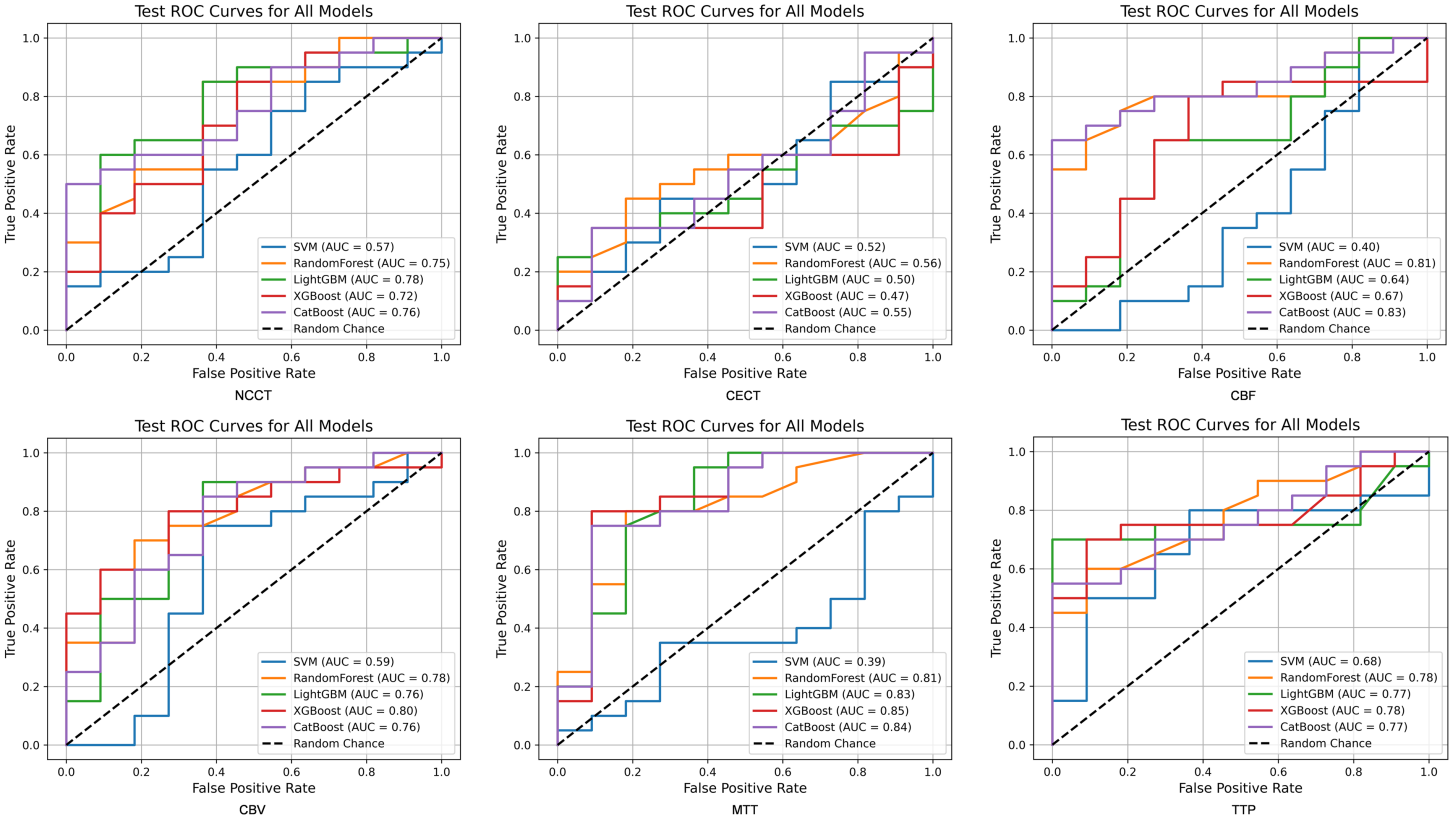
The performance of single-modality CT radiomics models.

Among six single-modality CT models, perfusion (CTP) maps outperformed structural CT (NCCT) for outcome discrimination, while CECT consistently underperformed. For NCCT, the most effective model (LightGBM) achieved an AUC of 0.782, with an accuracy of 0.774, sensitivity of 0.850, specificity of 0.636, and F1 score of 0.829. CECT produced AUCs < 0.60 in all algorithms (best: Random Forest AUC = 0.564). Given its uniformly poor performance, CECT was not considered in subsequent imaging-only fusions. In terms of CTP, the modalities incorporating CBF and MTT exhibited the highest efficiency. The CBF model (CatBoost) achieved an AUC of 0.832 (accuracy = 0.710, sensitivity = 0.800, specificity = 0.545, F1 = 0.780), while the XGBoost model using MTT attained an AUC of 0.855 (accuracy = 0.806, sensitivity = 0.850, specificity = 0.727, F1 = 0.850) (Table 2; Figure 5).

**Table 2:** Results of single-modality CT radiomics models.

Modality	Best algorithm	Accuracy	Precision	Recall	Specificity	F1 score	AUC
NCCT	LightGBM	0.774	0.810	0.850	0.636	0.829	0.782
CECT	Random forest	0.548	0.667	0.600	0.455	0.632	0.564
CBF	CatBoost	0.710	0.762	0.800	0.545	0.780	0.832
CBV	XGBoost	0.742	0.773	0.850	0.545	0.810	0.795
MTT	XGBoost	0.806	0.850	0.850	0.727	0.85	0.855
TTP	Random forest	0.710	0.739	0.850	0.455	0.791	0.782

### 3.3 Multi-modalities CT radiomics models

**Figure 6:**
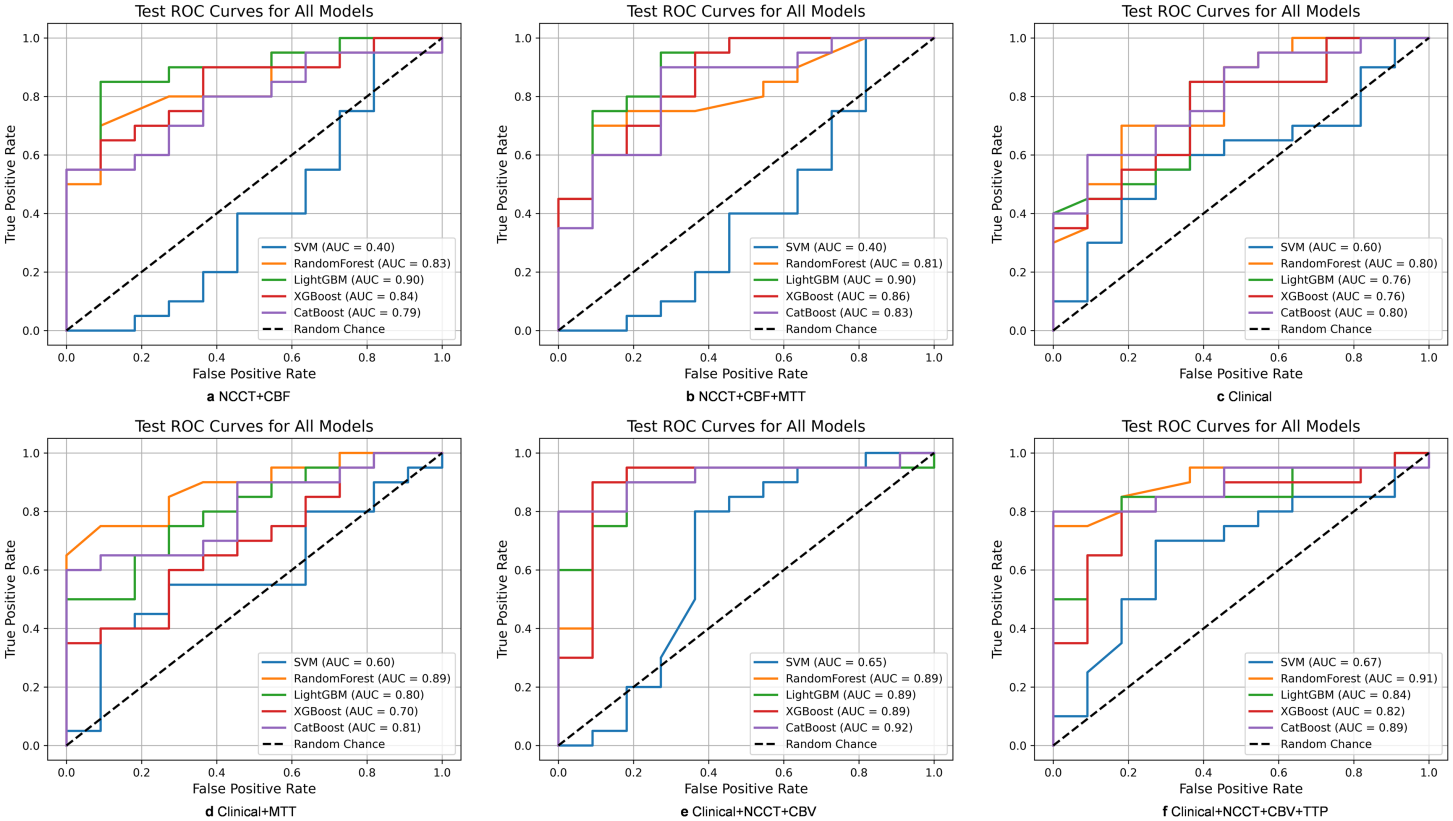
The best performance of Multi-modalities CT radiomics models and multimodal radiomics-clinical ensemble models, as well as the results of clinical models. **(a)** NCCT + CBF (LightGBM) performed best among dual-modality models; **(b)** NCCT + CBF + MTT (LightGBM) performed best among tri-modality models; **(c)** The performance of clinical models; **(d)** Clinical + MTT (Random Forest) performed best among clinical + single CT modality; **(e)** Clinical + NCCT + CBV (CatBoost) performed best among clinical + two CT modalities; **(f)** Clinical + NCCT + CBV + TTP (Random Forest) performed best among clinical + three CT modalities.

Fusing modalities improved performance relative to single-modality models, with the magnitude dependent on the complementarity of paired or triplet inputs. Among dual-modality models, NCCT+CBF (LightGBM) performed best with AUC = 0.895 (accuracy = 0.774, sensitivity = 0.900, specificity = 0.545, F1 = 0.837). Tri-modality fusion yielded additional gains in selected combinations. The top tri-modality model was NCCT+CBF + MTT (LightGBM) with AUC = 0.900, accuracy = 0.839, sensitivity = 0.900, specificity = 0.727, and F1 = 0.878 (Table 3; Figures 6a,b; Supplementary Figures 1–8; Supplementary Tables 1–8).

**Table 3:** Results of multi-modalities CT radiomics models.

Combination	Best algorithm	Accuracy	Precision	Recall	Specificity	F1 score	AUC
NCCT + CBF	LightGBM	0.774	0.783	0.900	0.545	0.837	0.895
NCCT + CBV	XGBoost	0.774	0.810	0.850	0.636	0.829	0.832
NCCT + MTT	LightGBM	0.742	0.833	0.750	0.727	0.789	0.855
NCCT + TTP	Random forest	0.677	0.708	0.850	0.364	0.773	0.836
NCCT + CBF + MTT	LightGBM	0.839	0.857	0.900	0.727	0.878	0.900
NCCT + CBV + MTT	LightGBM	0.871	0.864	0.950	0.727	0.905	0.886
NCCT + CBF + TTP	LightGBM	0.710	0.762	0.800	0.545	0.780	0.855
NCCT + CBV + TTP	Random forest	0.806	0.792	0.950	0.545	0.864	0.864

### 3.4 Clinical models

Using five clinically selected variables (Cys-C, lymphocyte, baseline NIHSS, onset-to-recanalization, ASPECTS), the clinical-only models demonstrated moderate performance, with the highest AUC reaching 0.800 (CatBoost). This model achieved an accuracy of 0.742, sensitivity of 0.900, specificity of 0.455, and an F1 score of 0.818. The Random Forest model showed comparable results with an AUC of 0.798 (Table 4; Figure 6c).

**Table 4:** Results of clinical models.

Algorithm	Accuracy	Precision	Recall	Specificity	F1 Score	AUC
SVM	0.548	0.800	0.400	0.818	0.533	0.600
Random forest	0.742	0.773	0.850	0.545	0.810	0.798
LightGBM	0.710	0.739	0.850	0.455	0.791	0.757
XGBoost	0.710	0.739	0.850	0.455	0.791	0.759

### 3.5 Multimodal radiomics-clinical ensemble models

The integration of clinical variables with radiomics consistently enhanced model performance compared to either radiomics-only or clinical-only approaches. For the clinical plus single radiomics modality, the optimal results were observed with the clinical + NCCT (CatBoost) model, which attained an AUC of 0.886 (accuracy = 0.839, sensitivity = 0.950, specificity = 0.636, F1 = 0.884). This outcome was superior to both the single clinical model (AUC = 0.800) and the NCCT-only model (AUC = 0.782). Following the combination of clinical and CTP modalities, the AUC for the Random Forest model using clinical + MTT rose to 0.889, marking an improvement over the single clinical model (AUC = 0.800) and the MTT-only model (AUC = 0.855). Notably, the ensemble model integrating clinical, NCCT, and CTP modalities–the clinical + NCCT + CBV model (CatBoost) –achieved the highest performance in the study, with an AUC of 0.918, accuracy of 0.806, sensitivity of 0.950, specificity of 0.545, and F1 score of 0.864. Additional pairings also improved over their radiomics-only counterparts, e.g., clinical+NCCT+TTP (Random Forest) AUC = 0.907 and clinical+NCCT+CBF (Random Forest) model reached an AUC of 0.855. However, when combining clinical variables with three imaging modalities, the results were mixed and did not consistently surpass the best dual-fusion models (Table 5; Figures 6d–f; Supplementary Figures 9–22; Supplementary Tables 9–22).

**Table 5:** Results of multimodal radiomics-clinical ensemble models.

Combination	Best algorithm	Accuracy	Precision	Recall	Specificity	F1 Score	AUC
Clinical + NCCT	CatBoost	0.839	0.826	0.950	0.636	0.884	0.886
Clinical + CECT	Random forest	0.806	0.850	0.850	0.727	0.850	0.830
Clinical + CBF	CatBoost	0.742	0.750	0.900	0.455	0.818	0.841
Clinical + CBV	CatBoost	0.806	0.818	0.900	0.636	0.857	0.882
Clinical + MTT	Random forest	0.742	0.750	0.900	0.455	0.818	0.889
Clinical + TTP	CatBoost	0.742	0.750	0.900	0.455	0.818	0.873
Clinical + NCCT + CBF	Random forest	0.774	0.783	0.900	0.545	0.837	0.855
Clinical + NCCT + CBV	CatBoost	0.806	0.792	0.950	0.545	0.864	0.918
Clinical + NCCT + MTT	Random forest	0.774	0.760	0.950	0.455	0.844	0.848
Clinical + NCCT + TTP	Random forest	0.774	0.760	0.950	0.455	0.844	0.907
Clinical + NCCT + CBF + MTT	Random forest	0.774	0.760	0.950	0.455	0.844	0.905
Clinical + NCCT + CBV + MTT	LightGBM	0.742	0.731	0.950	0.364	0.826	0.855
Clinical + NCCT + CBF + TTP	Random forest	0.806	0.818	0.900	0.636	0.857	0.891
Clinical + NCCT + CBV + TTP	Random forest	0.774	0.760	0.950	0.455	0.844	0.907

### 3.6 Classifier performance comparison

We assessed five distinct machine learning classifiers (SVM, Random Forest, LightGBM, XGBoost, and CatBoost) to identify the most effective model for our prediction task. Overall, ensemble tree-based models, particularly the gradient boosting algorithms, exhibited a performance advantage over SVM and standard Random Forest classifiers. XGBoost and CatBoost consistently achieved the highest AUCs across most feature sets. The Random Forest classifier performed adequately but lagged behind the boosting models. The SVM classifier displayed more variability in its results. LightGBM remained consistently competitive, often finishing within a narrow margin of the leading models and occasionally best-in-class for select dual- and tri-modality fusions (e.g., NCCT+CBF, NCCT+CBF + MTT)–but on average trailed XGBoost/CatBoost by a small margin (Supplementary Figures 1–22; Supplementary Tables 1–22).

## 4 Discussion

FR presents a significant challenge in the context of EVT and there is a lack of reliable predictive models for assessing treatment outcomes in acute ischemic stroke. In this study, we employ machine learning techniques to combine multimodal CT radiomics, clinical, laboratory, and angiographic data for accurate prediction of FR. Ultimately, our multimodal CT radiomics-clinical ensemble machine learning model with the CatBoost algorithm demonstrates excellent performance, achieving an AUC of 0.918.

Among single CT modality models, CTP modalities exhibited the best performance, followed by NCCT, while CECT underperformed. The superior performance of CTP compared to NCCT can be attributed to its ability to provide comprehensive hemodynamic information regarding the ischemic brain tissues (Saver et al., 2015). Brugnara et al. (2020) suggested that the predictive value of CTP for FR is limited, potentially because their research focused solely on the infarct core and ischemic penumbra, rather than conducting a comprehensive radiomics analysis of individual CTP parameters. The limited contrast resolution of enhanced scans may also hinder feature discrimination in CECT. Utilizing an anatomy-function complementarity strategy, the performance of dual-modality (NCCT+CTP) models was enhanced. Furthermore, incorporating a third modality generally improved discrimination when it contributed non-redundant hemodynamic information. Among tri-modality CT radiomics, the optimal combined model was NCCT + CBF + MTT, achieving an AUC of 0.900, with the most significant gains observed when pairing NCCT with both a volume (CBV) and a time (MTT/TTP) parameter. This finding indicates that the integrated analysis of structural maps along with cerebral blood volume and hemodynamic information over time is crucial for prognostic predictions.

In the preliminary univariate analysis of clinical variables, age, baseline NIHSS, Cys-C, the time from onset to puncture and other factors were significantly associated with functional outcomes at 90 days. Most predictors align with previous studies, though some discrepancies may arise from differences in research subjects and sample sizes. Notably, consistent findings across studies (including ours) reveal that older age, higher preoperative mRS and NIHSS scores, elevated NLR, lower ASPECTS and GCS scores, and prolonged time from onset to puncture and recanalization are associated with FR (Nie et al., 2023; D’Anna et al., 2024; Deng et al., 2022; Ni et al., 2023; Shen et al., 2023). These associations are also clinically plausible. Older age and higher baseline disability may reflect reduced physiological and neurological reserve, while higher NIHSS and lower GCS are markers of more severe initial neurological injury. Lower ASPECTS generally indicates a larger extent of early ischemic change, and elevated NLR may reflect a stronger systemic inflammatory response that contributes to secondary injury. In addition, longer treatment delays may increase irreversible tissue damage before reperfusion is achieved, thereby increasing the likelihood of poor functional recovery despite technically successful recanalization. Overall, the performance of clinical models lagged behind that of CT radiomics models. This discrepancy may be due to clinical variables being more susceptible to variability and possessing lower specificity. Among the clinical models, CatBoost and Random Forest demonstrated the best comprehensive performance, with AUC of 0.800 and 0.798, respectively. This suggests that these machine learning algorithms are well-suited as prognostic prediction tools based on clinical information, providing a reliable baseline for subsequent ensemble modeling with CT radiomics data.

When integrating clinical indicators with each CT radiomics modality for modeling, the ensemble model generally outperformed both the single clinical and CT radiomics models, underscoring the advantages of ensemble modeling. Remarkably, the clinical + NCCT + CBV ensemble model using CatBoost achieved an AUC of 0.918, surpassing any single modality or other combined models. This highlights the potential of the multimodal omics integration strategy in predicting treatment efficacy after endovascular interventions, thereby supporting the pursuit of more precise individualized treatment. However, results for the clinical + three CT modalities results were mixed and did not consistently surpass the best tri-fusion models. Consequently, integrating more modalities does not always guarantee superior outcomes. Given the inherent correlations among CTP parameters, adding more modalities generally improves performance when the added maps provide non-redundant hemodynamic information. Collectively, fusion models centered on NCCT + CBV (with or without a time parameter) and enhanced by clinical variables yielded the most robust discrimination.

In this study, gradient boosting algorithms (especially XGBoost and CatBoost) showed a performance advantage over SVM and standard Random Forest across most feature sets. LightGBM consistently demonstrated competitive performance, often within a narrow margin of the top performers and occasionally excelling in selected dual- and tri-modality fusions. This finding aligns with previous studies on stroke outcome prediction (Mbarek et al., 2024). The superior performance of boosted trees can be attributed to their capability to model complex non-linear feature interactions and manage high-dimensional data with inherent regularization (shrinkage and tree pruning), making them well-suited for radiomics features. While the Random Forest classifier performed reasonably well, it lagged behind the boosting models, likely due to its inability to focus on the most informative features as effectively as gradient boosting, which iteratively minimizes residual errors. The SVM classifier displayed more variable results in our overall comparison. SVM tended to be more sensitive to the curse of dimensionality. Its performance was slightly lower with full feature sets and required careful tuning of hyperparameters (kernel parameters and regularization) to prevent overfitting. In contrast, tree-based models managed large feature pools more robustly by automatically selecting splits on the most predictive features. LightGBM utilized histogram-based leaf-wise growth with built-in regularization. These findings highlight the importance of algorithm selection in integrated multimodal studies, as ensemble learning methods can unlock more prognostic signals within the data, while simpler classifiers may underfit or overfit if not matched to the feature characteristics. Thus, fusing multiple classifiers or conducting a rigorous model selection process is recommended to ensure optimal predictive performance for a given radiomics application.

Our study has several limitations. First, the relatively limited sample size and retrospective single-center design may restrict the generalizability of the models, because institutional differences in imaging workflow, clinical decision-making, and laboratory testing may affect model applicability in other settings. Further prospective studies with larger multicenter samples are needed to validate these findings externally. Second, postoperative CT and clinical data were not included in this study. Integrating preoperative and postoperative data may further enhance predictive performance. Third, our analysis combined AIS with anterior and posterior circulation occlusions, because these subgroups may differ in pathophysiology, treatment response, and clinical prognosis, this combined analysis may have introduced heterogeneity. Future studies with larger cohorts should analyze these populations separately.

## 5 Conclusion

In conclusion, the multimodal CT radiomics-clinical ensemble model effectively predicts FR following EVT for AIS using gradient boosting algorithms. The ensemble model, when appropriately tailored with relevant variables and algorithms, has the potential to facilitate personalized treatment and improve clinical outcomes for AIS.

## Data Availability

The original contributions presented in the study are included in the article/Supplementary material, further inquiries can be directed to the corresponding author.
