# Supplementary material for: Multimodal CT radiomics-clinical ensemble machine learning model effectively predicts futile recanalization after endovascular treatment of acute ischemic stroke
Source: Front Neurosci. 2026 May 12;20:1838675. doi: 10.3389/fnins.2026.1838675 (PMC13201450; doi:10.3389/fnins.2026.1838675)
Supplement: Supplementary file 1 [file Data_Sheet_1.PDF]

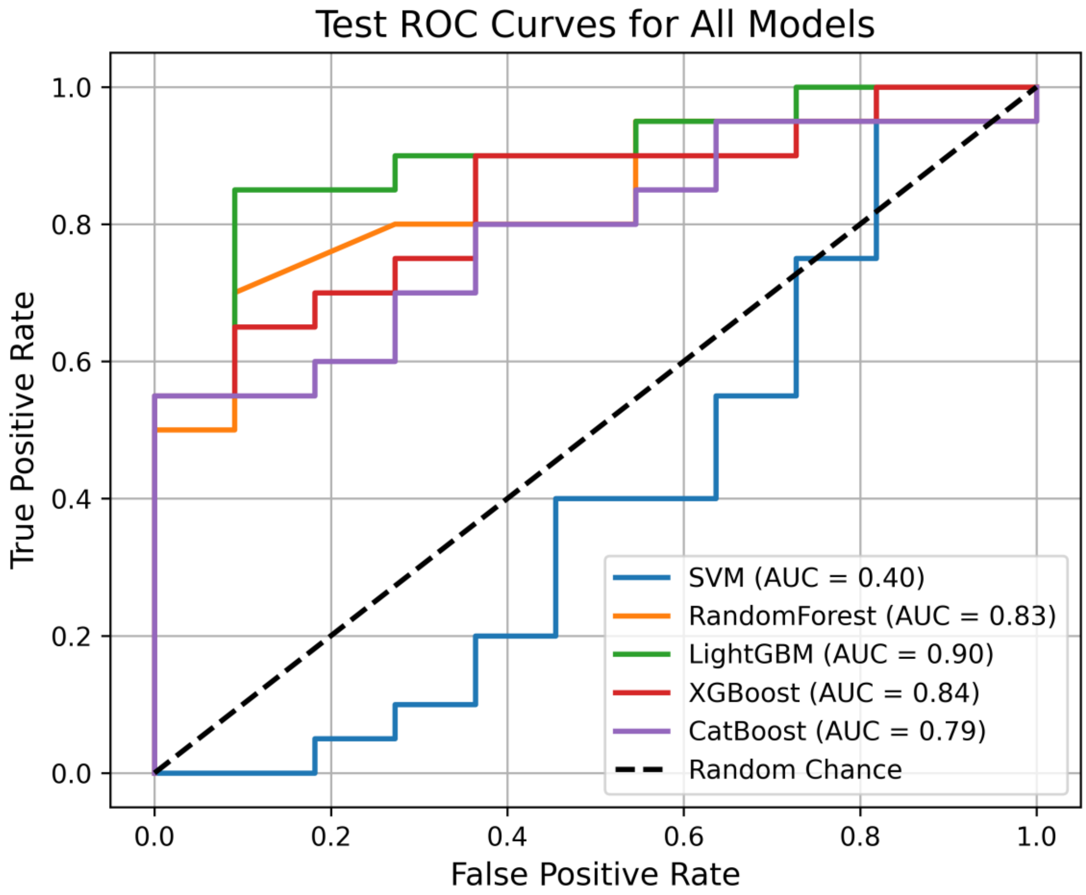

Supplemental Fig. 1 The performance of NCCT + CBF Model.

Supplemental Table 1 Results of NCCT + CBF Model

NCCT + CBF Model	Accuracy	Precision	Recall	Specificity	F1 Score	AUC
SVM	0.677	0.679	0.950	0.182	0.792	0.405
RandomForest	0.710	0.762	0.800	0.545	0.780	0.832
LightGBM	0.774	0.783	0.900	0.545	0.837	0.895
XGBoost	0.742	0.800	0.800	0.636	0.800	0.836
CatBoost	0.710	0.739	0.850	0.455	0.791	0.786

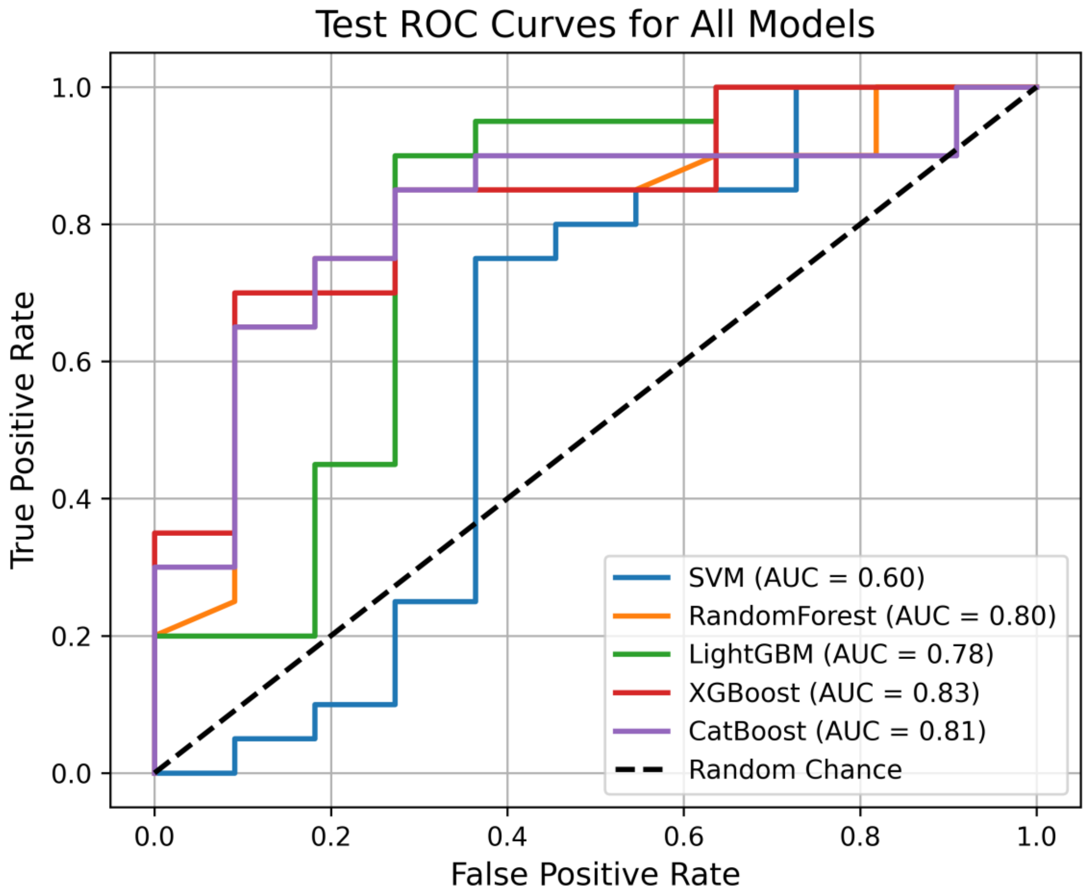

Supplemental Fig. 2 The performance of NCCT + CBV Model.

Supplemental Table 2 Results of NCCT + CBV Model

NCCT + CBV Model	Accuracy	Precision	Recall	Specificity	F1 Score	AUC
SVM	0.677	0.692	0.900	0.273	0.783	0.605
RandomForest	0.774	0.810	0.850	0.636	0.829	0.805
LightGBM	0.806	0.818	0.900	0.636	0.857	0.782
XGBoost	0.774	0.810	0.850	0.636	0.829	0.832
CatBoost	0.806	0.818	0.900	0.636	0.857	0.814

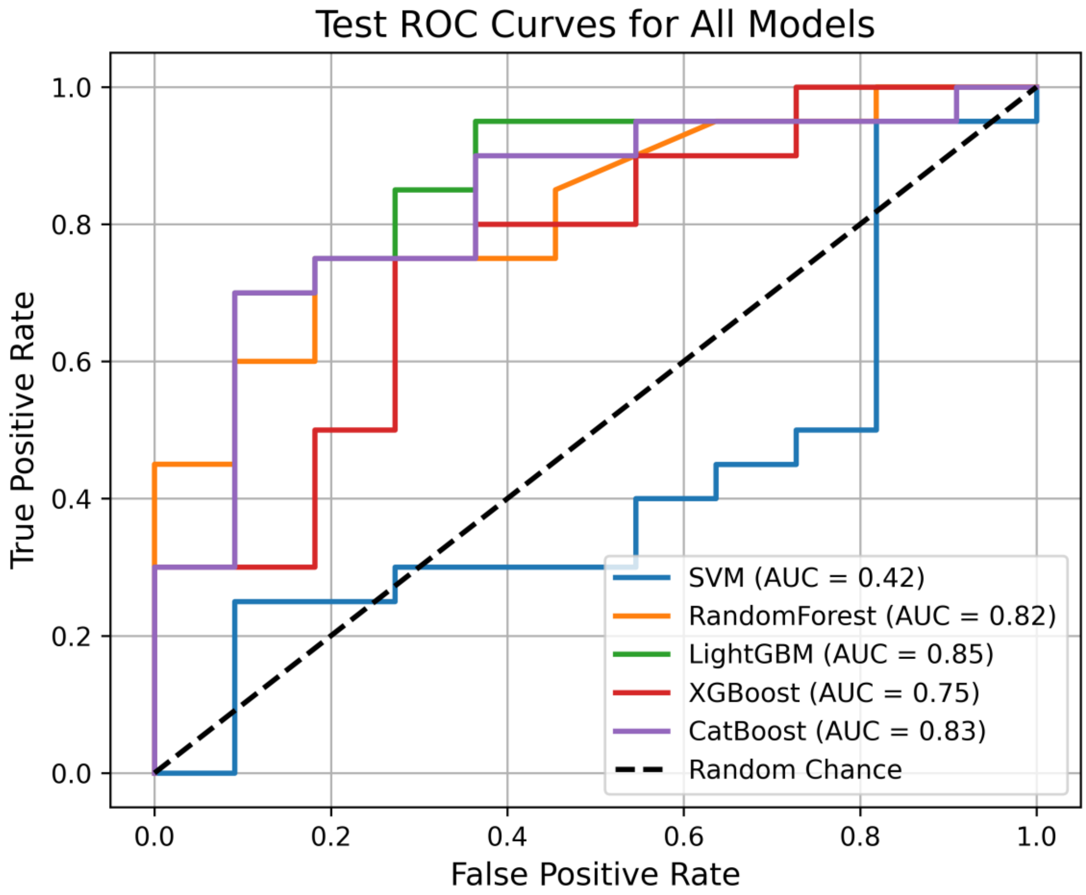

Supplemental Fig. 3 The performance of NCCT + MTT Model.

Supplemental Table 3 Results of NCCT + MTT Model

NCCT + MTT Model	Accuracy	Precision	Recall	Specificity	F1 Score	AUC
SVM	0.613	0.654	0.850	0.182	0.739	0.423
RandomForest	0.677	0.750	0.750	0.545	0.750	0.818
LightGBM	0.742	0.833	0.750	0.727	0.789	0.855
XGBoost	0.677	0.727	0.800	0.455	0.762	0.750
CatBoost	0.774	0.810	0.850	0.636	0.829	0.827

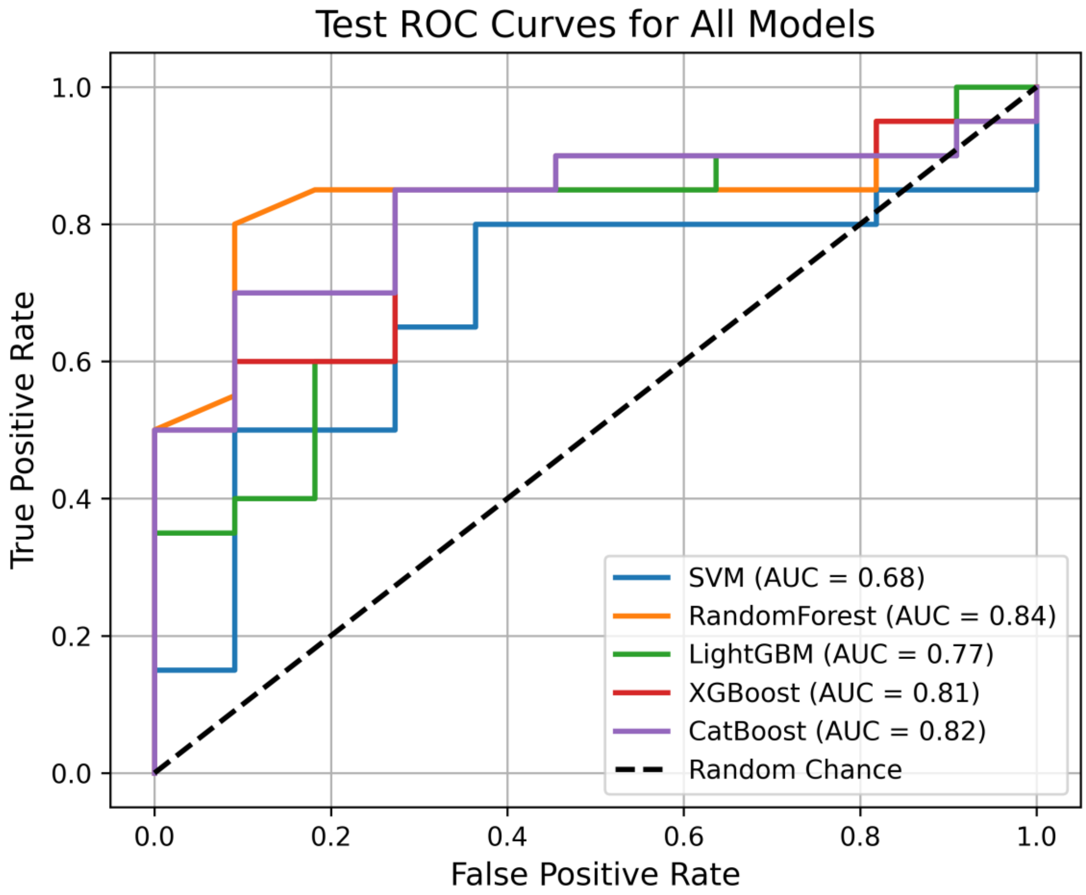

Supplemental Fig. 4 The performance of NCCT + TTP Model.

Supplemental Table 4 Results of NCCT + TTP Model

NCCT + TTP Model	Accuracy	Precision	Recall	Specificity	F1 Score	AUC
SVM	0.452	0.800	0.200	0.909	0.320	0.682
RandomForest	0.677	0.708	0.850	0.364	0.773	0.836
LightGBM	0.710	0.739	0.850	0.455	0.791	0.768
XGBoost	0.742	0.773	0.850	0.545	0.810	0.809
CatBoost	0.677	0.692	0.900	0.273	0.783	0.823

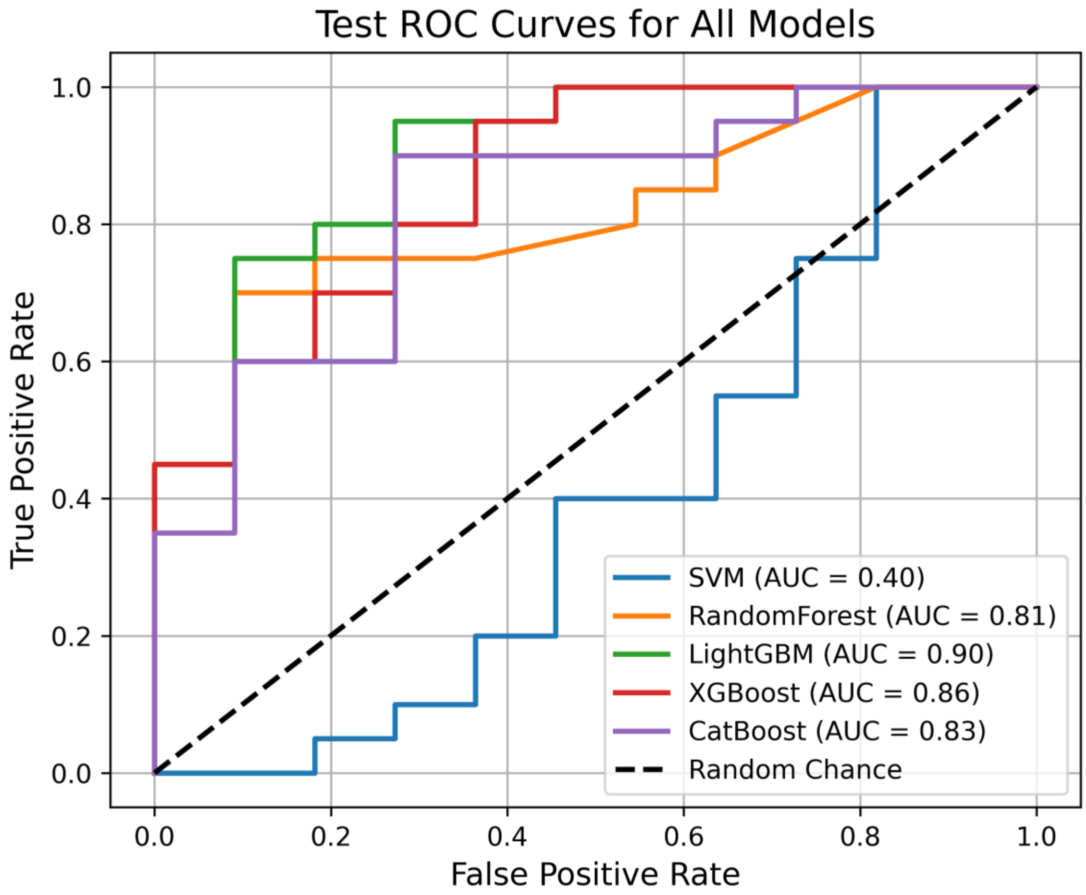

Supplemental Fig. 5 The performance of NCCT + CBF + MTT Model.

Supplemental Table 5 Results of NCCT + CBF + MTT Model

NCCT + CBF + MTT Model	Accuracy	Precision	Recall	Specificity	F1 Score	AUC
SVM	0.677	0.679	0.950	0.182	0.792	0.405
RandomForest	0.710	0.789	0.750	0.636	0.769	0.814
LightGBM	0.839	0.857	0.900	0.727	0.878	0.900
XGBoost	0.742	0.800	0.800	0.636	0.800	0.864
CatBoost	0.774	0.842	0.800	0.727	0.821	0.827

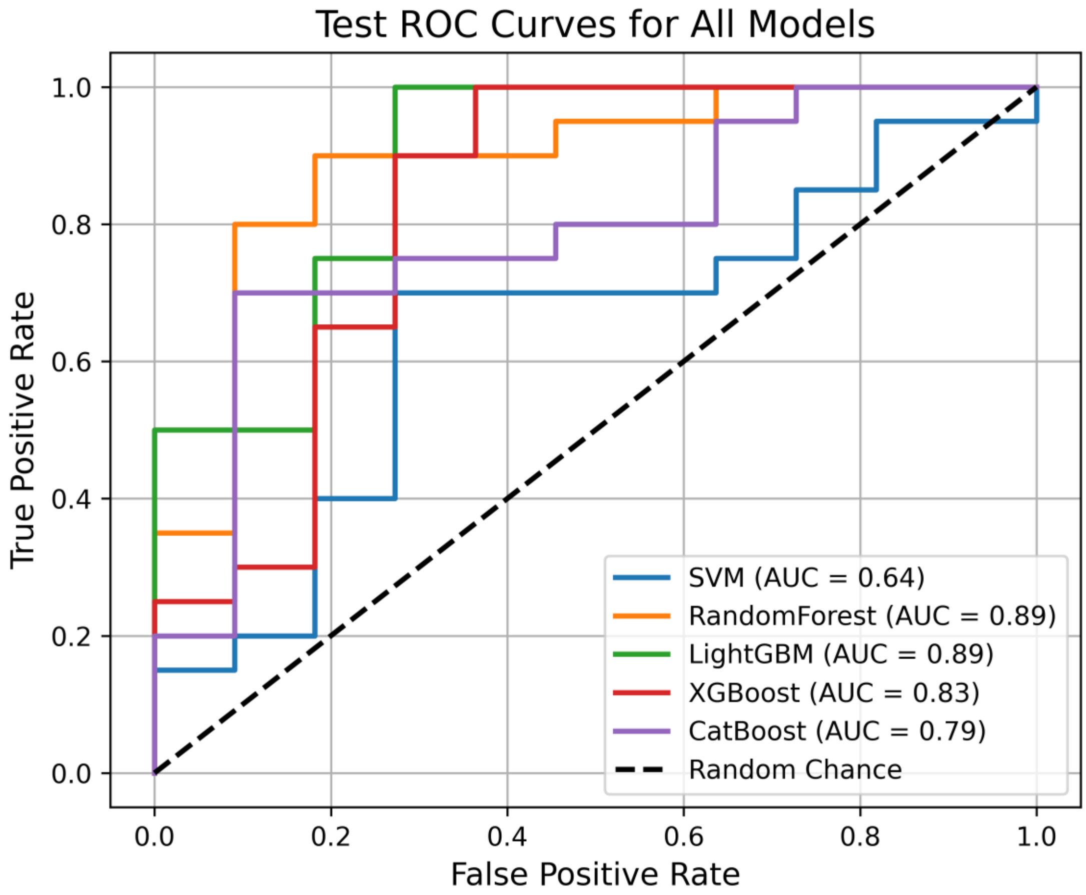

Supplemental Fig. 6 The performance of NCCT + CBV + MTT Model.

Supplemental Table 6 Results of NCCT + CBV + MTT Model

NCCT + CBV + MTT Model	Accuracy	Precision	Recall	Specificity	F1 Score	AUC
SVM	0.581	0.652	0.750	0.273	0.698	0.641
RandomForest	0.839	0.857	0.900	0.727	0.878	0.886
LightGBM	0.871	0.864	0.950	0.727	0.905	0.886
XGBoost	0.839	0.826	0.950	0.636	0.884	0.827
CatBoost	0.677	0.708	0.850	0.364	0.773	0.786

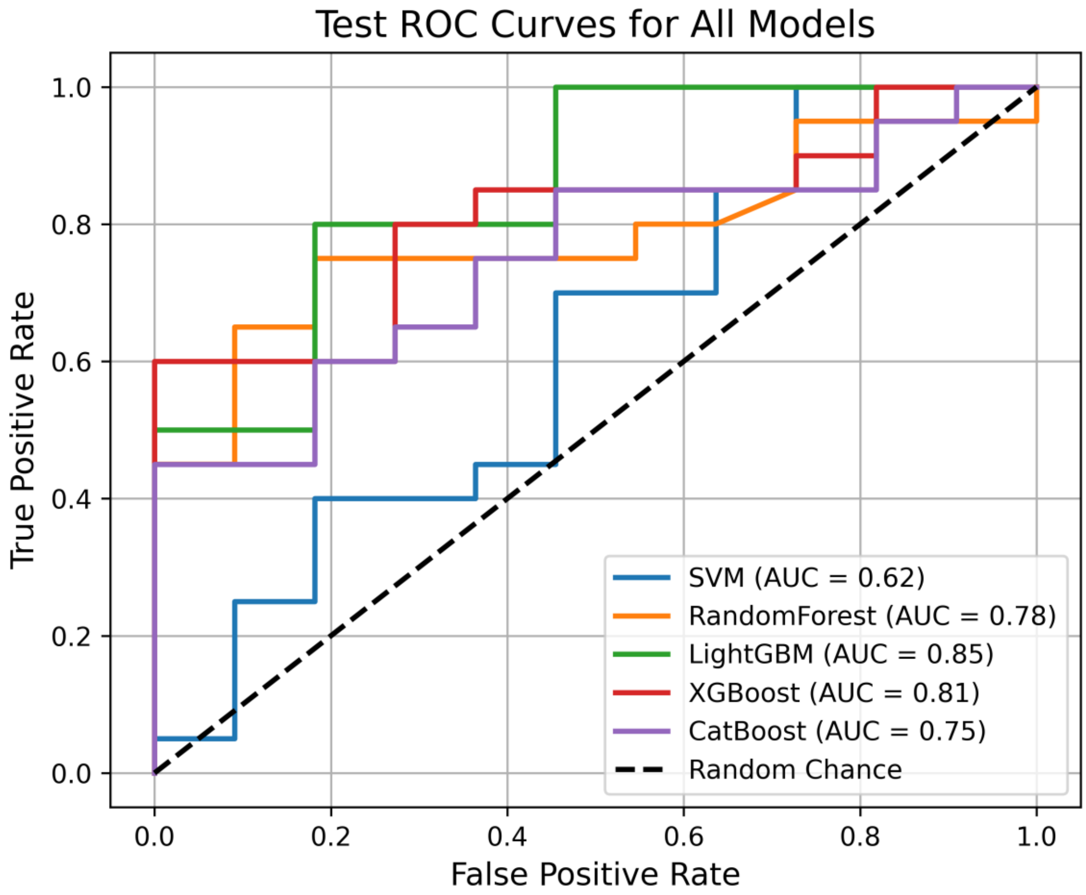

Supplemental Fig. 7 The performance of NCCT + CBF + TTP Model.

Supplemental Table 7 Results of NCCT + CBF + TTP Model

NCCT+CBF+TTP Model	Accuracy	Precision	Recall	Specificity	F1 Score	AUC
SVM	0.645	0.696	0.800	0.364	0.744	0.618
RandomForest	0.645	0.696	0.800	0.364	0.744	0.780
LightGBM	0.710	0.762	0.800	0.545	0.780	0.855
XGBoost	0.774	0.810	0.850	0.636	0.829	0.809
CatBoost	0.677	0.708	0.850	0.364	0.773	0.750

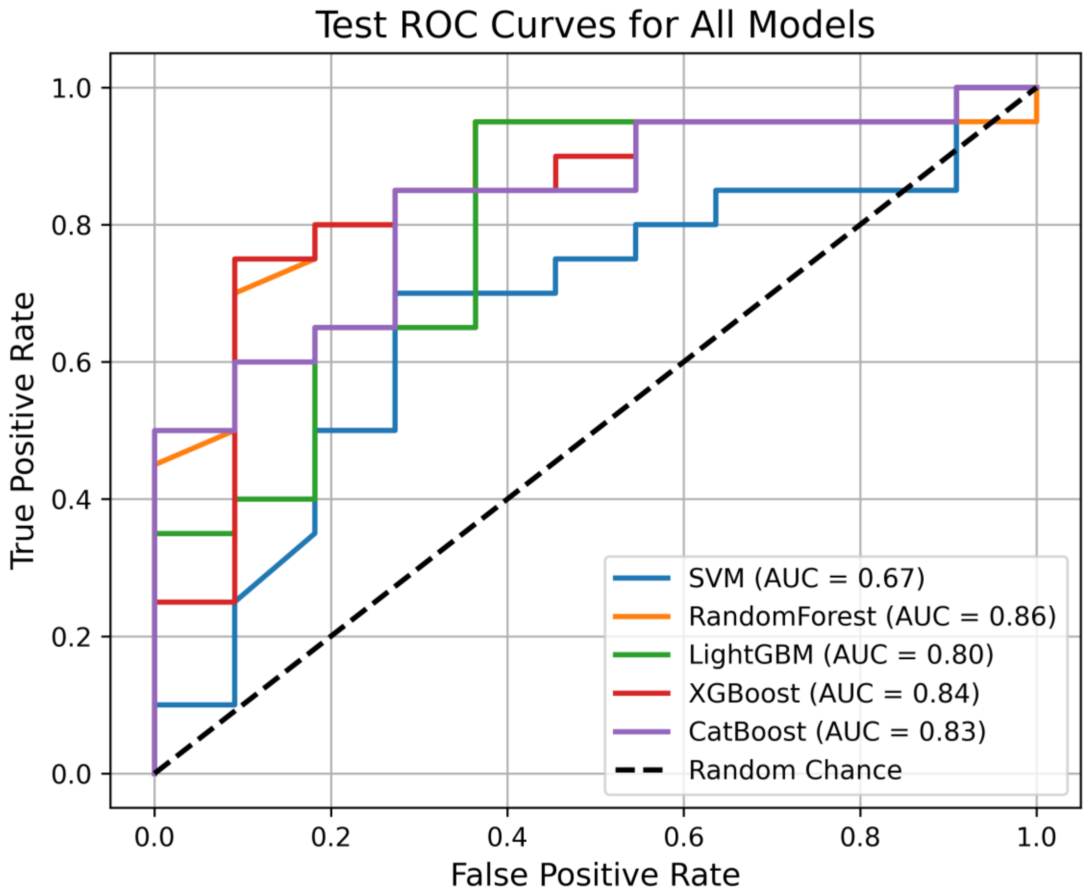

Supplemental Fig. 8 The performance of NCCT + CBV + TTP Model.

Supplemental Table 8 Results of NCCT + CBV + TTP Model

NCCT + CBV + TTP Model	Accuracy	Precision	Recall	Specificity	F1 Score	AUC
SVM	0.484	0.833	0.250	0.909	0.385	0.673
RandomForest	0.806	0.792	0.950	0.545	0.864	0.864
LightGBM	0.806	0.792	0.950	0.545	0.864	0.795
XGBoost	0.742	0.750	0.900	0.455	0.818	0.836
CatBoost	0.742	0.750	0.900	0.455	0.818	0.827

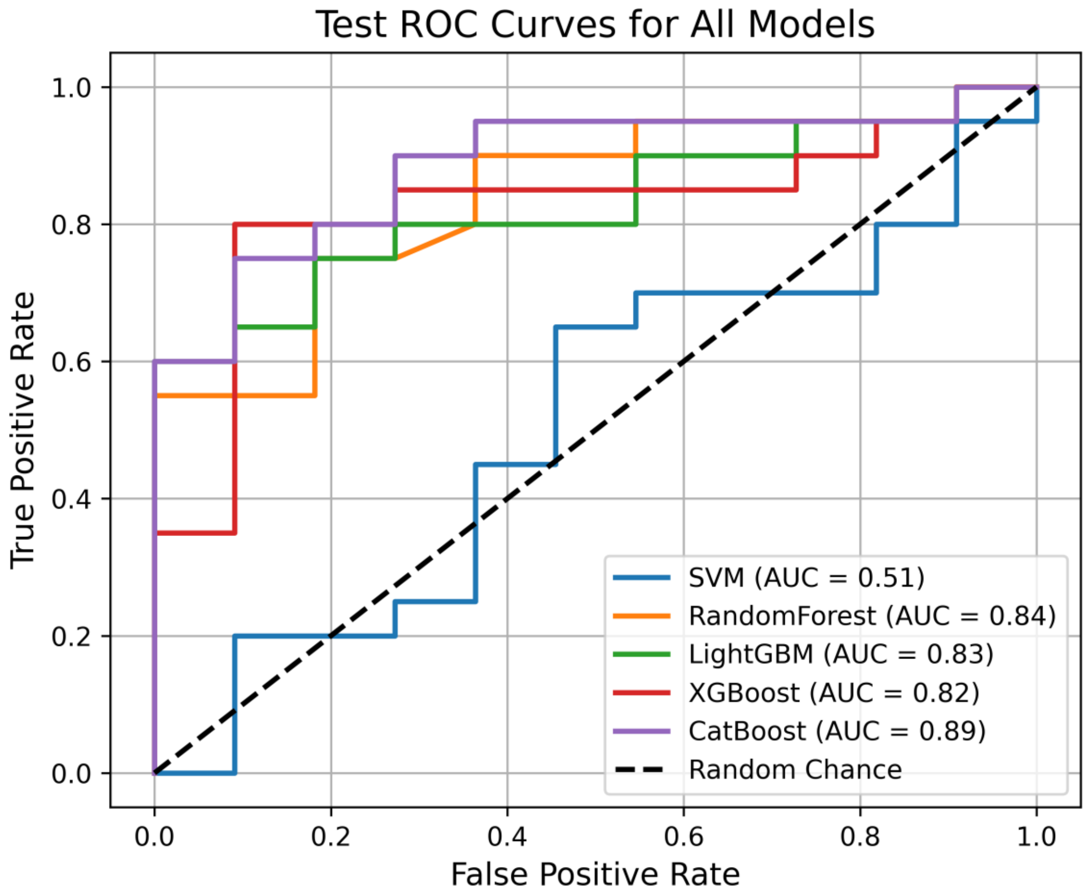

Supplemental Fig. 9 The performance of Clinical + NCCT Model.

Supplemental Table 9 Results of Clinical + NCCT Model

Clinical + NCCT Model	Accuracy	Precision	Recall	Specificity	F1 Score	AUC
SVM	0.484	0.667	0.400	0.636	0.500	0.509
RandomForest	0.742	0.750	0.900	0.455	0.818	0.839
LightGBM	0.677	0.679	0.950	0.182	0.792	0.827
XGBoost	0.645	0.680	0.850	0.273	0.756	0.823
CatBoost	0.839	0.826	0.950	0.636	0.884	0.886

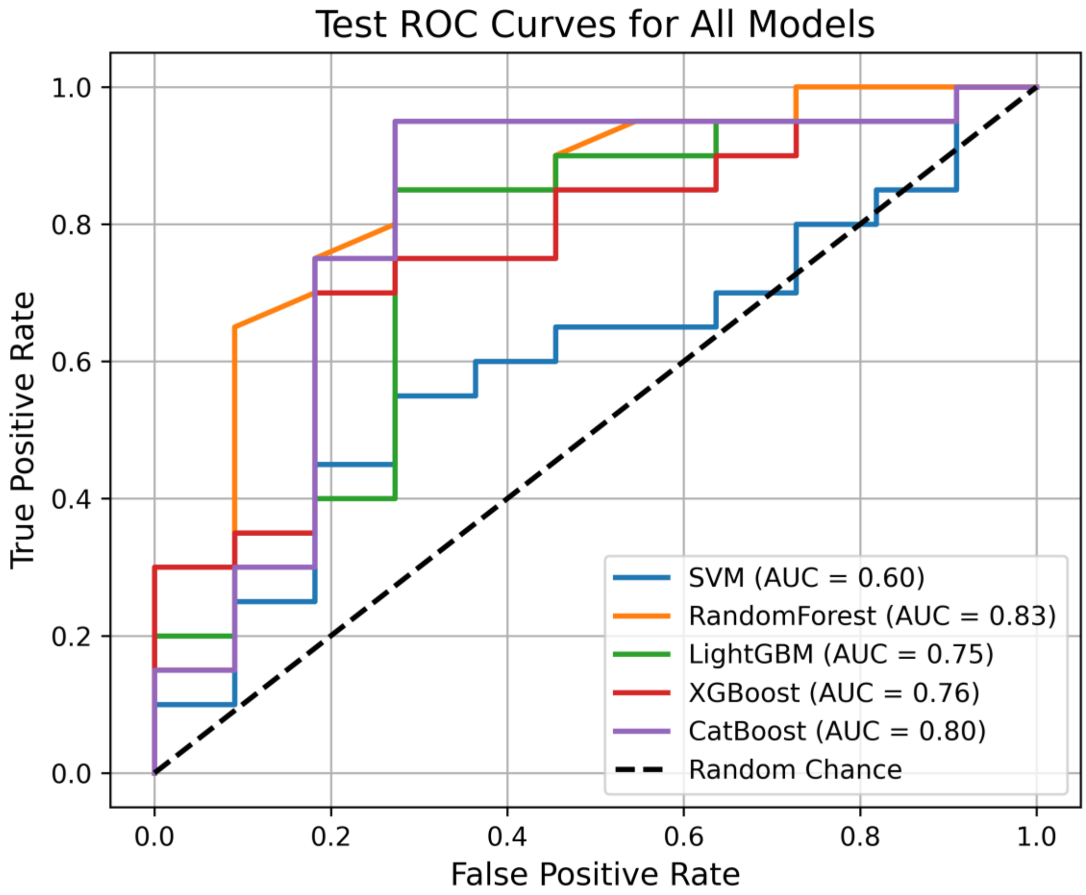

Supplemental Fig. 10 The performance of Clinical + CECT Model.

Supplemental Table 10 Results of Clinical + CECT Model

Clinical + CECT Model	Accuracy	Precision	Recall	Specificity	F1 Score	AUC
SVM	0.548	0.750	0.450	0.727	0.563	0.600
RandomForest	0.806	0.850	0.850	0.727	0.850	0.830
LightGBM	0.742	0.773	0.850	0.545	0.810	0.755
XGBoost	0.710	0.789	0.750	0.636	0.769	0.759
CatBoost	0.839	0.857	0.900	0.727	0.878	0.805

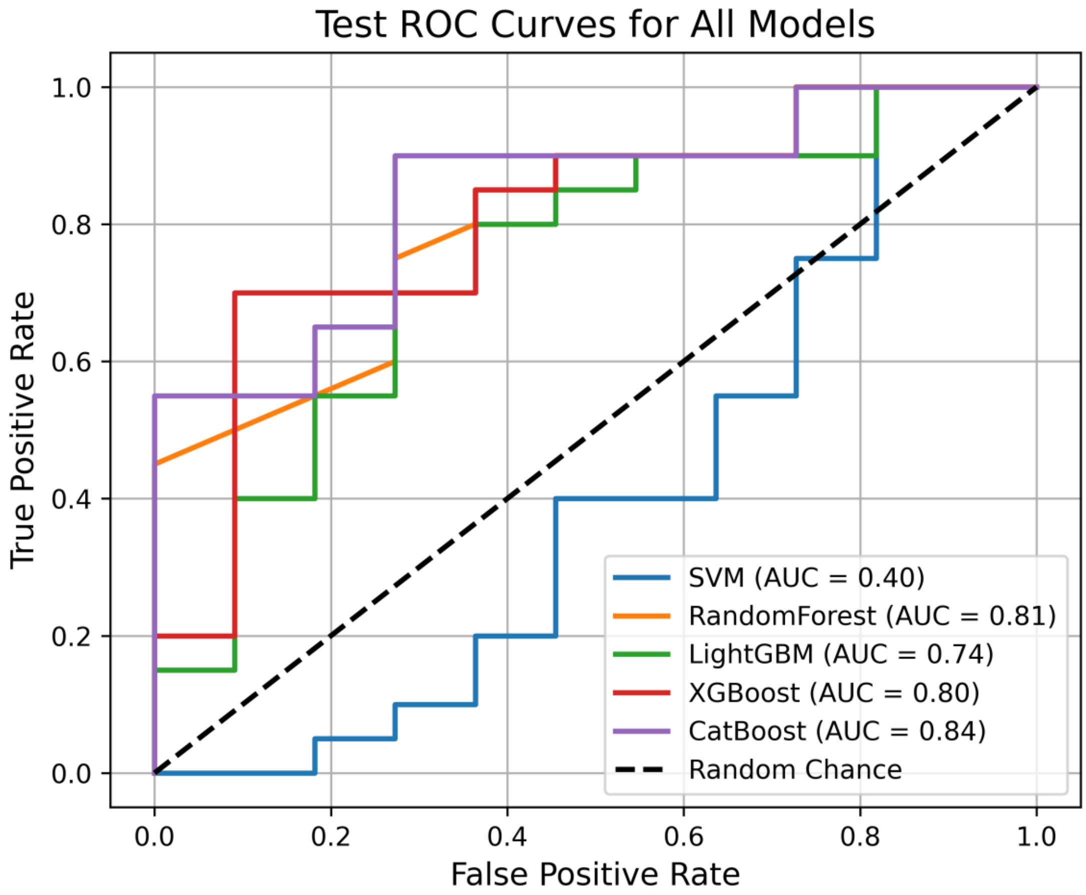

Supplemental Fig. 11 The performance of Clinical + CBF Model.

Supplemental Table 11 Results of Clinical + CBF Model

Clinical + CBF Model	Accuracy	Precision	Recall	Specificity	F1 Score	AUC
SVM	0.677	0.679	0.950	0.182	0.792	0.405
RandomForest	0.742	0.773	0.850	0.545	0.810	0.809
LightGBM	0.742	0.773	0.850	0.545	0.810	0.741
XGBoost	0.710	0.720	0.900	0.364	0.800	0.805
CatBoost	0.742	0.750	0.900	0.455	0.818	0.841

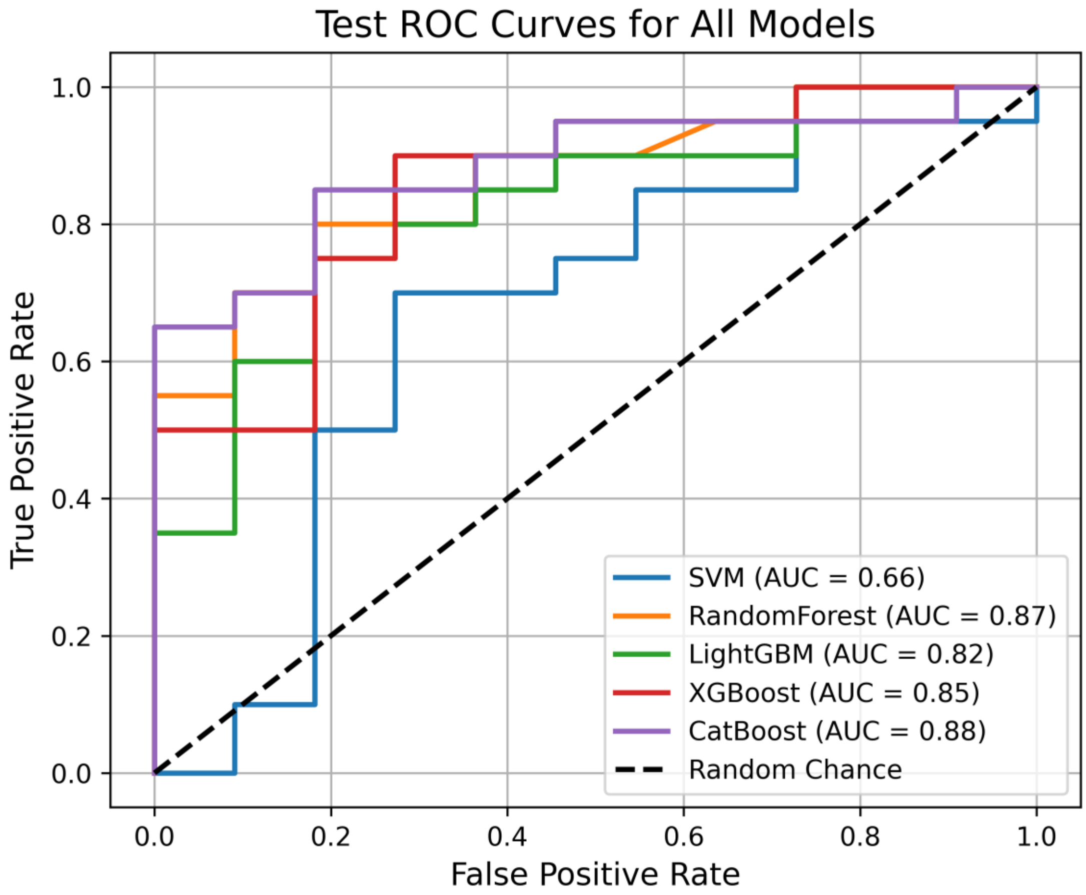

Supplemental Fig. 12 The performance of Clinical + CBV Model.

Supplemental Table 12 Results of Clinical + CBV Model

Clinical + CBV Model	Accuracy	Precision	Recall	Specificity	F1 Score	AUC
SVM	0.645	0.800	0.600	0.727	0.686	0.664
RandomForest	0.806	0.818	0.900	0.636	0.857	0.866
LightGBM	0.710	0.720	0.900	0.364	0.800	0.823
XGBoost	0.774	0.760	0.950	0.455	0.844	0.855
CatBoost	0.806	0.818	0.900	0.636	0.857	0.882

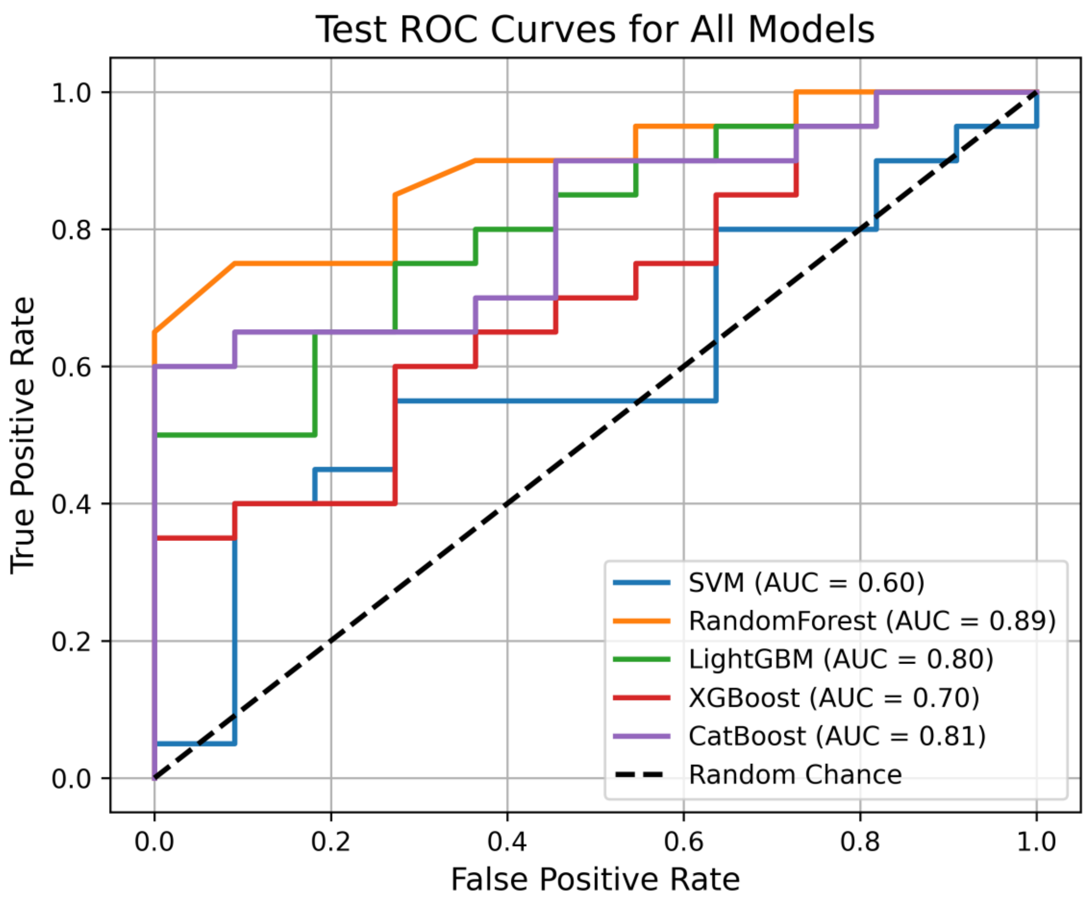

Supplemental Fig. 13 The performance of Clinical + MTT Model.

Supplemental Table 13 Results of Clinical + MTT Model

Clinical + MTT Model	Accuracy	Precision	Recall	Specificity	F1 Score	AUC
SVM	0.516	0.632	0.600	0.364	0.615	0.595
RandomForest	0.742	0.750	0.900	0.455	0.818	0.889
LightGBM	0.710	0.739	0.850	0.455	0.791	0.805
XGBoost	0.613	0.682	0.750	0.364	0.714	0.695
CatBoost	0.742	0.750	0.900	0.455	0.818	0.809

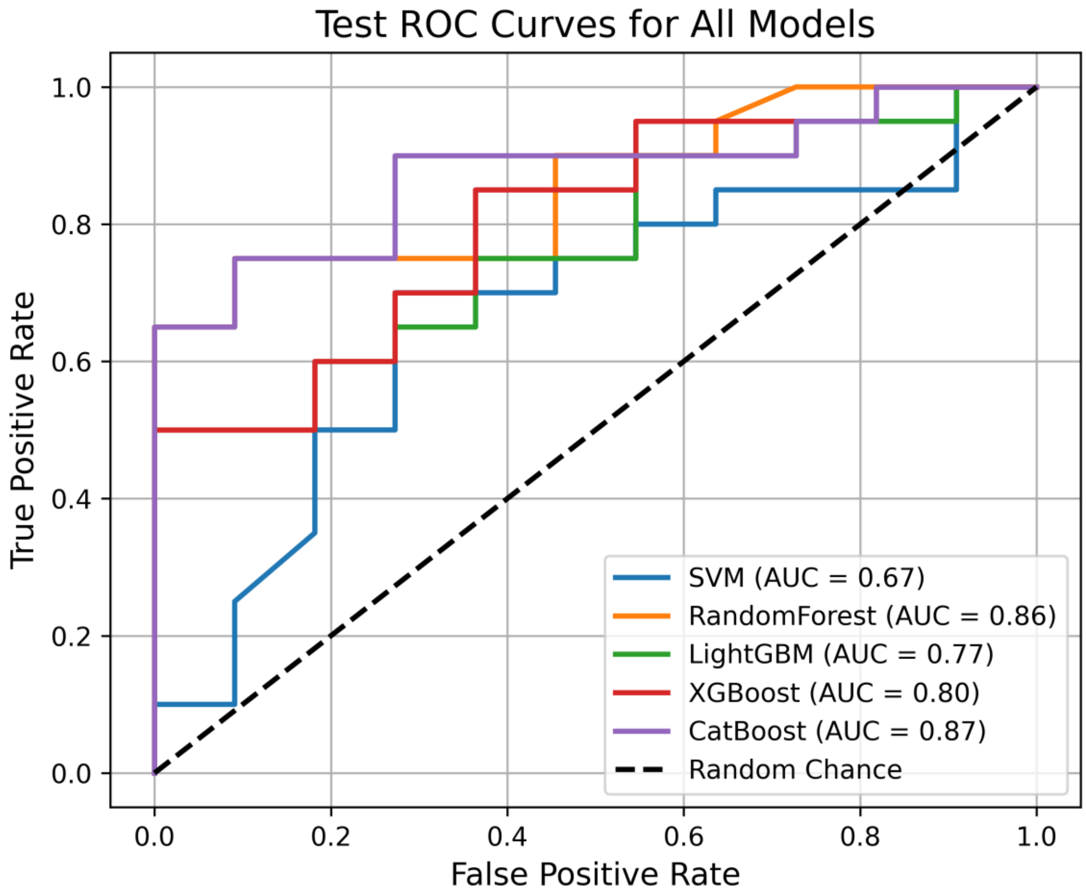

Supplemental Fig. 14 The performance of Clinical + TTP Model.

Supplemental Table 14 Results of Clinical + TTP Model

Clinical + TTP Model	Accuracy	Precision	Recall	Specificity	F1 Score	AUC
SVM	0.484	0.833	0.250	0.909	0.385	0.673
RandomForest	0.774	0.783	0.900	0.545	0.837	0.857
LightGBM	0.710	0.720	0.900	0.364	0.800	0.768
XGBoost	0.774	0.760	0.950	0.455	0.844	0.805
CatBoost	0.742	0.750	0.900	0.455	0.818	0.873

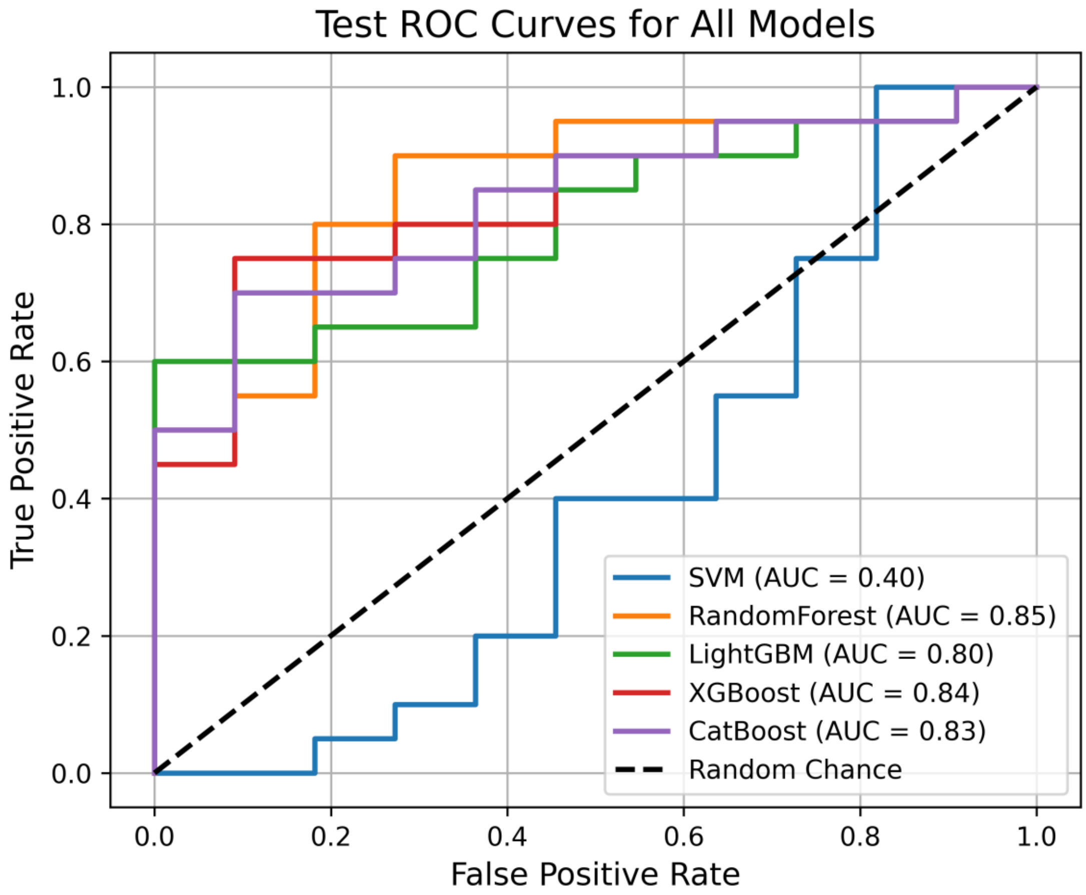

Supplemental Fig. 15 The performance of Clinical + NCCT + CBF Model.

Supplemental Table 15 Results of Clinical + NCCT+ CBF Model

Clinical + NCCT + CBF Model	Accuracy	Precision	Recall	Specificity	F1 Score	AUC
SVM	0.677	0.679	0.950	0.182	0.792	0.405
RandomForest	0.774	0.783	0.900	0.545	0.837	0.855
LightGBM	0.677	0.692	0.900	0.273	0.783	0.800
XGBoost	0.742	0.750	0.900	0.455	0.818	0.836
CatBoost	0.710	0.720	0.900	0.364	0.800	0.832

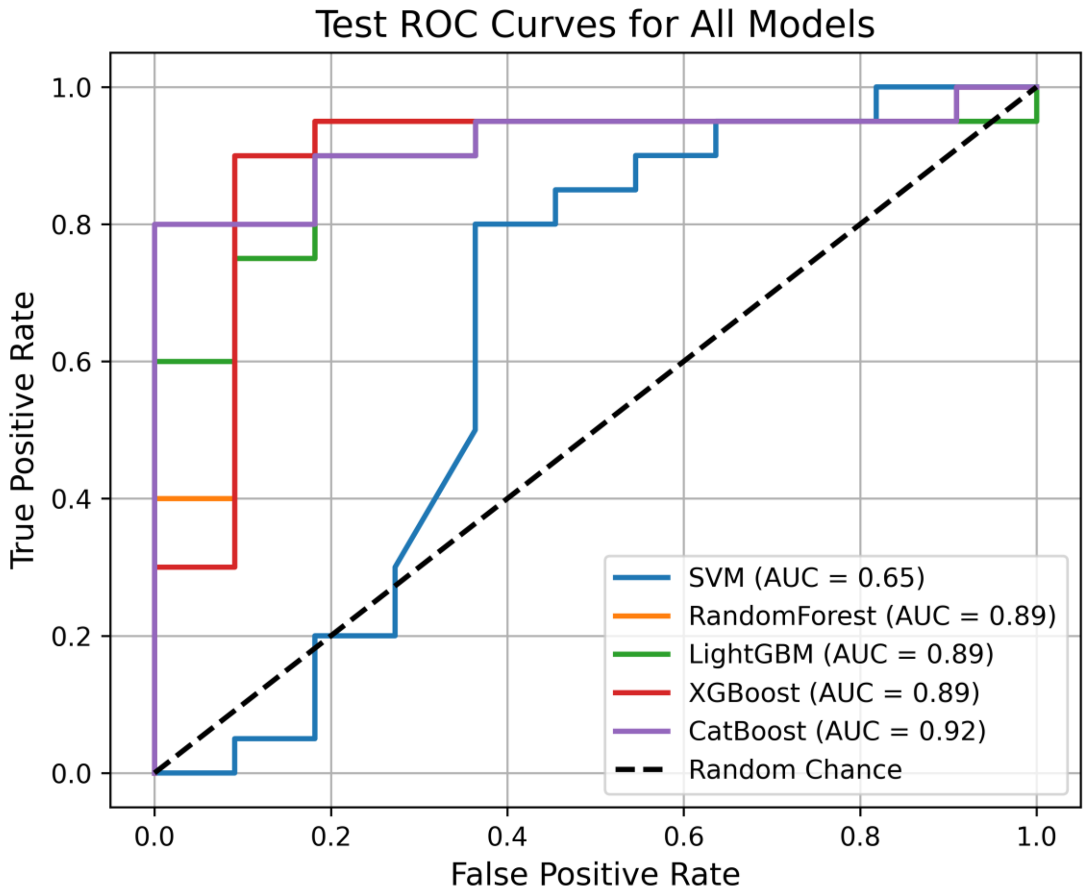

Supplemental Fig. 16 The performance of Clinical + NCCT + CBV Model.

Supplemental Table 16 Results of Clinical + NCCT+ CBV Model

Clinical + NCCT + CBV Model	Accuracy	Precision	Recall	Specificity	F1 Score	AUC
SVM	0.645	0.765	0.650	0.636	0.703	0.645
RandomForest	0.806	0.792	0.950	0.545	0.864	0.886
LightGBM	0.710	0.704	0.950	0.273	0.809	0.891
XGBoost	0.742	0.731	0.950	0.364	0.826	0.891
CatBoost	0.806	0.792	0.950	0.545	0.864	0.918

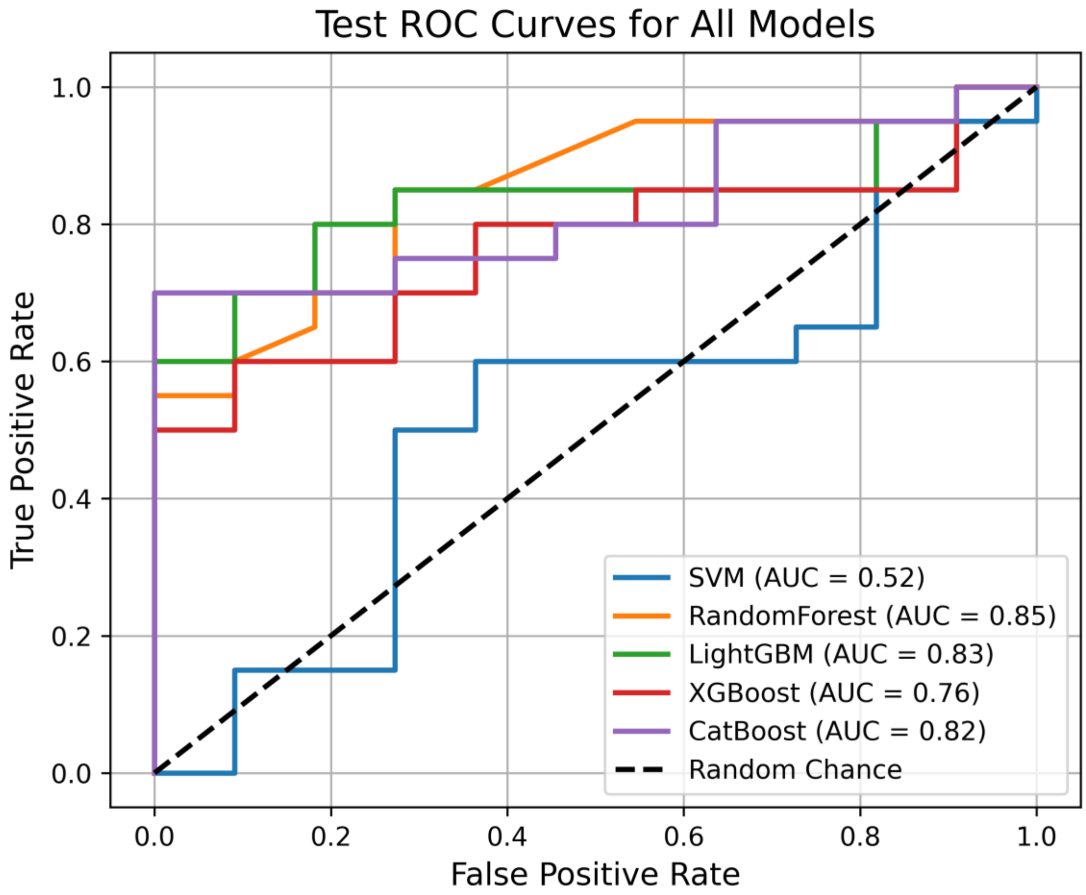

Supplemental Fig. 17 The performance of Clinical + NCCT + MTT Model.

Supplemental Table 17 Results of Clinical + NCCT+ MTT Model

Clinical + NCCT + MTT Model	Accuracy	Precision	Recall	Specificity	F1 Score	AUC
SVM	0.581	0.640	0.800	0.182	0.711	0.523
RandomForest	0.774	0.760	0.950	0.455	0.844	0.848
LightGBM	0.645	0.680	0.850	0.273	0.756	0.832
XGBoost	0.710	0.739	0.850	0.455	0.791	0.764
CatBoost	0.742	0.731	0.950	0.364	0.826	0.823

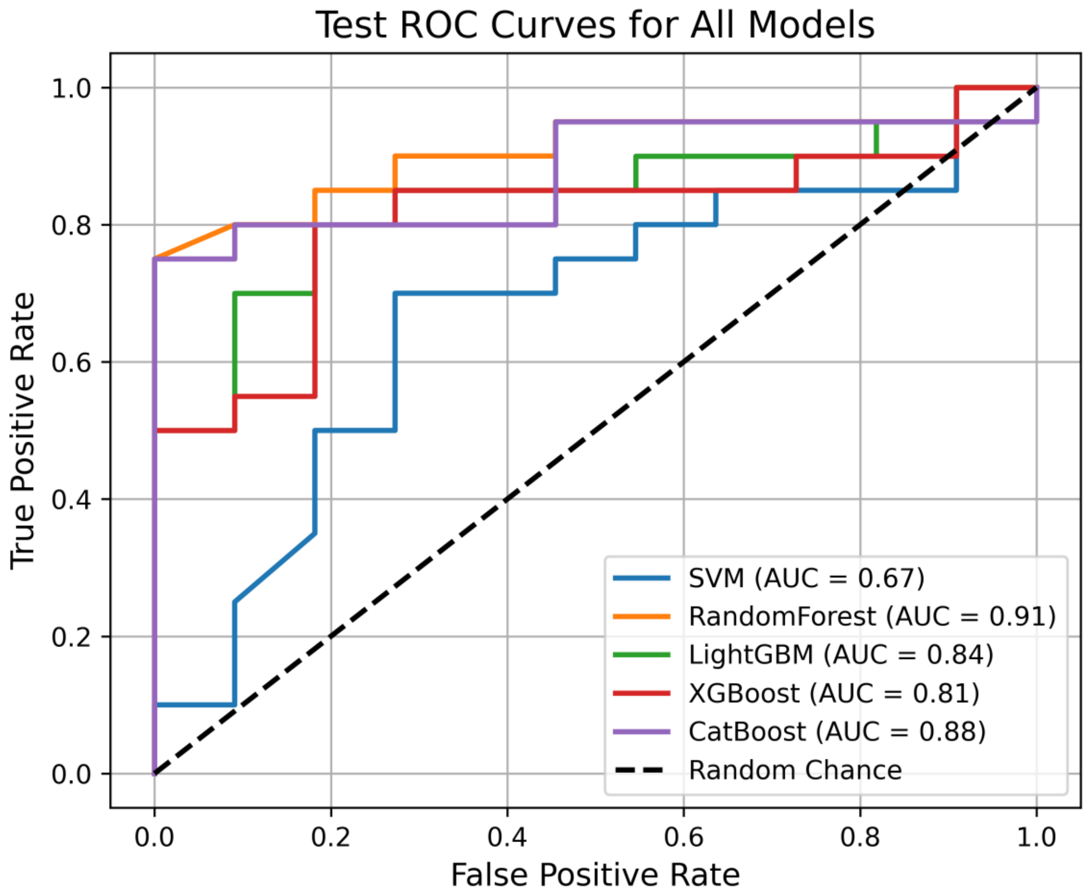

Supplemental Fig. 18 The performance of Clinical + NCCT + TTP Model.

Supplemental Table 18 Results of Clinical + NCCT+ TTP Model

Clinical + NCCT + TTP Model	Accuracy	Precision	Recall	Specificity	F1 Score	AUC
SVM	0.484	0.833	0.250	0.909	0.385	0.673
RandomForest	0.774	0.760	0.950	0.455	0.844	0.907
LightGBM	0.742	0.773	0.850	0.545	0.810	0.836
XGBoost	0.742	0.773	0.850	0.545	0.810	0.809
CatBoost	0.806	0.792	0.950	0.545	0.864	0.877

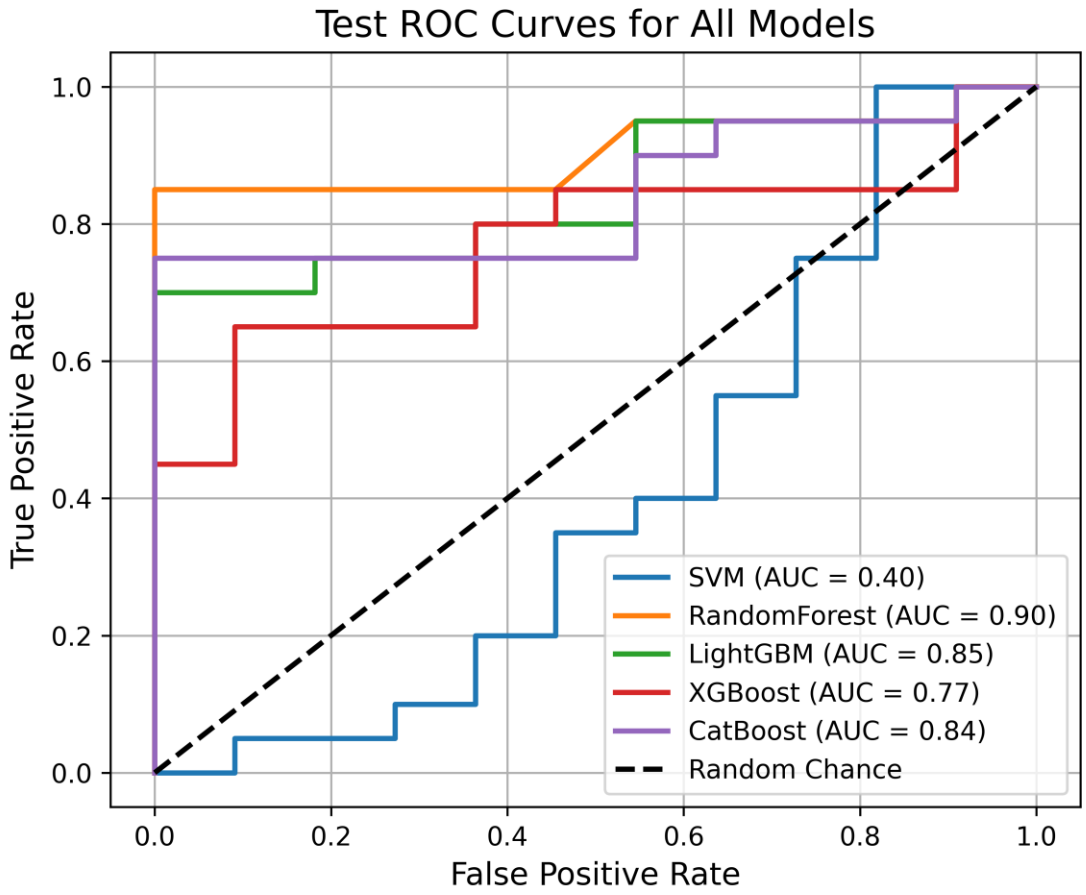

Supplemental Fig. 19 The performance of Clinical + NCCT + CBF + MTT Model.

Supplemental Table 19 Results of Clinical + NCCT+ CBF + MTT Model

Clinical + NCCT + CBF +MTT Model	Accuracy	Precision	Recall	Specificity	F1 Score	AUC
SVM	0.677	0.679	0.950	0.182	0.792	0.405
RandomForest	0.774	0.760	0.950	0.455	0.844	0.905
LightGBM	0.677	0.727	0.800	0.455	0.762	0.845
XGBoost	0.677	0.708	0.850	0.364	0.773	0.768
CatBoost	0.710	0.720	0.900	0.364	0.800	0.841

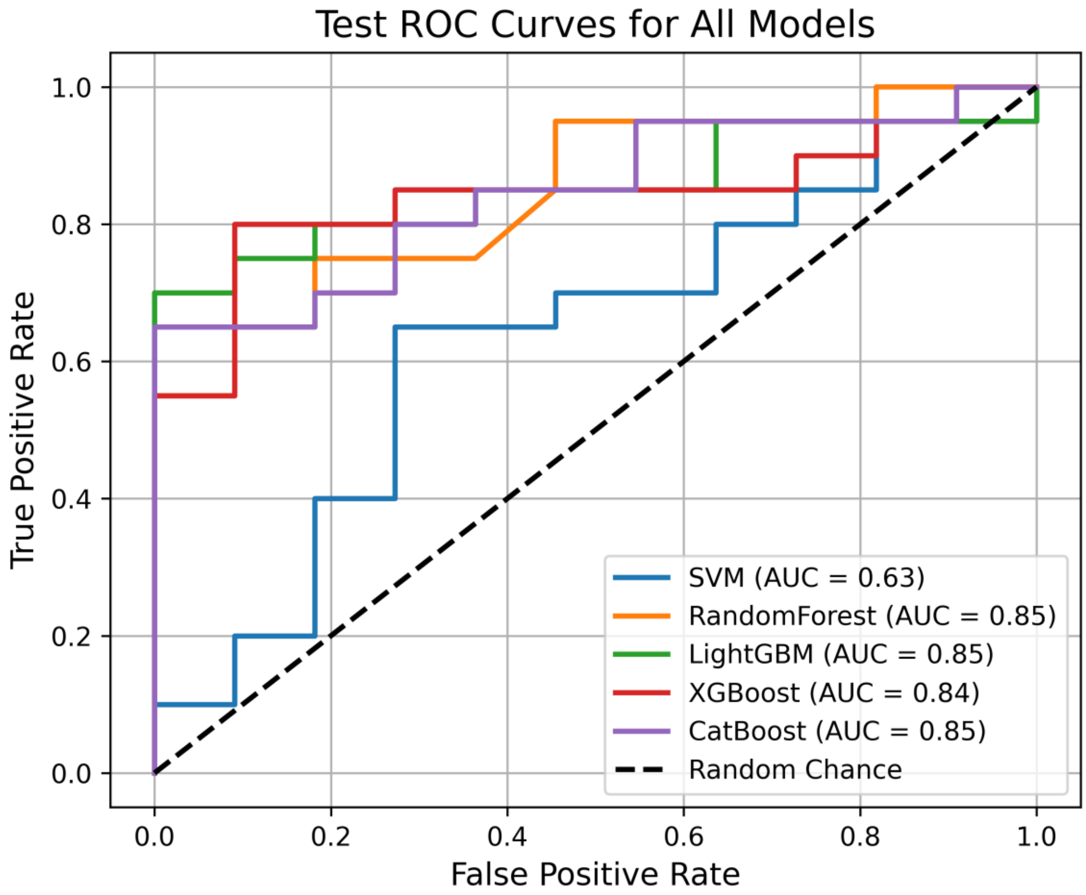

Supplemental Fig. 20 The performance of Clinical + NCCT + CBV + MTT Model.

Supplemental Table 20 Results of Clinical + NCCT+ CBV + MTT Model

Clinical + NCCT + CBV +MTT Model	Accuracy	Precision	Recall	Specificity	F1 Score	AUC
SVM	0.645	0.696	0.800	0.364	0.744	0.632
RandomForest	0.774	0.760	0.950	0.455	0.844	0.845
LightGBM	0.742	0.731	0.950	0.364	0.826	0.855
XGBoost	0.677	0.708	0.850	0.364	0.773	0.841
CatBoost	0.774	0.760	0.950	0.455	0.844	0.845

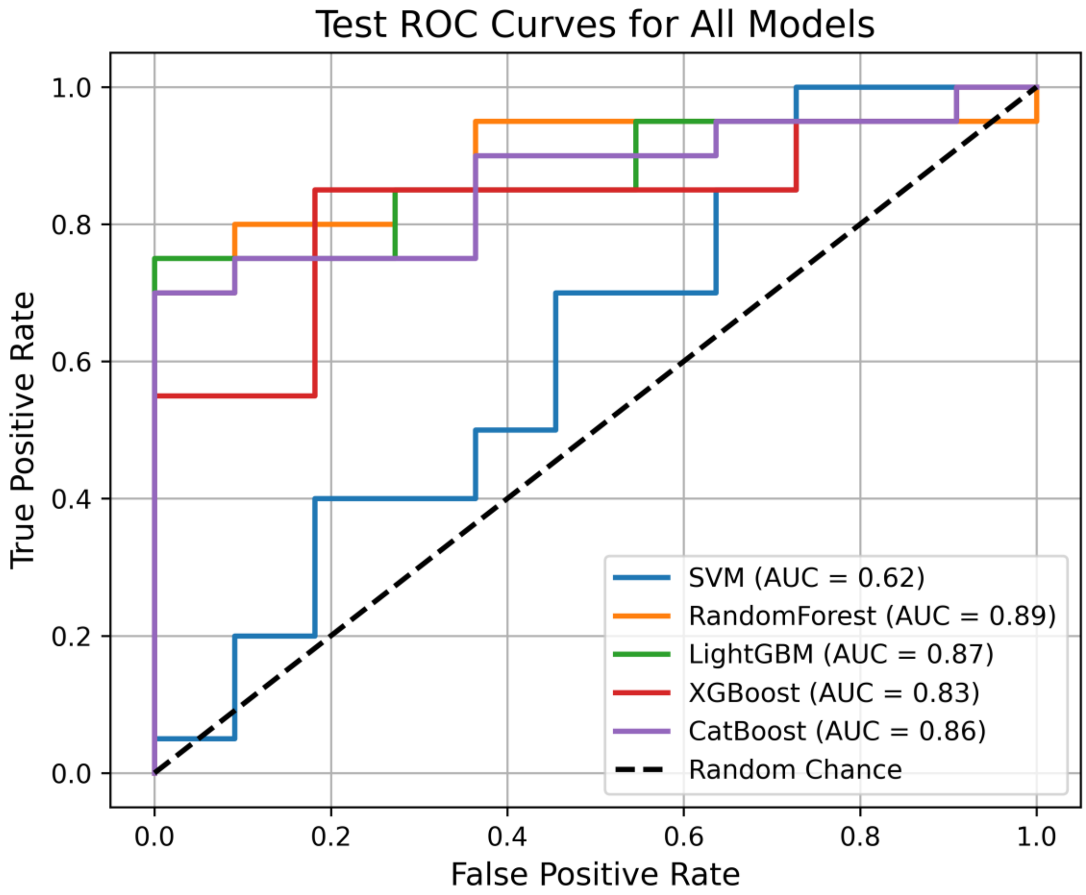

Supplemental Fig. 21 The performance of Clinical + NCCT + CBF + TTP Model.

Supplemental Table 21 Results of Clinical + NCCT+ CBF + TTP Model

Clinical + NCCT + CBF +TTP Model	Accuracy	Precision	Recall	Specificity	F1 Score	AUC
SVM	0.645	0.696	0.800	0.364	0.744	0.618
RandomForest	0.806	0.818	0.900	0.636	0.857	0.891
LightGBM	0.710	0.739	0.850	0.455	0.791	0.873
XGBoost	0.806	0.850	0.850	0.727	0.850	0.827
CatBoost	0.710	0.720	0.900	0.364	0.800	0.864

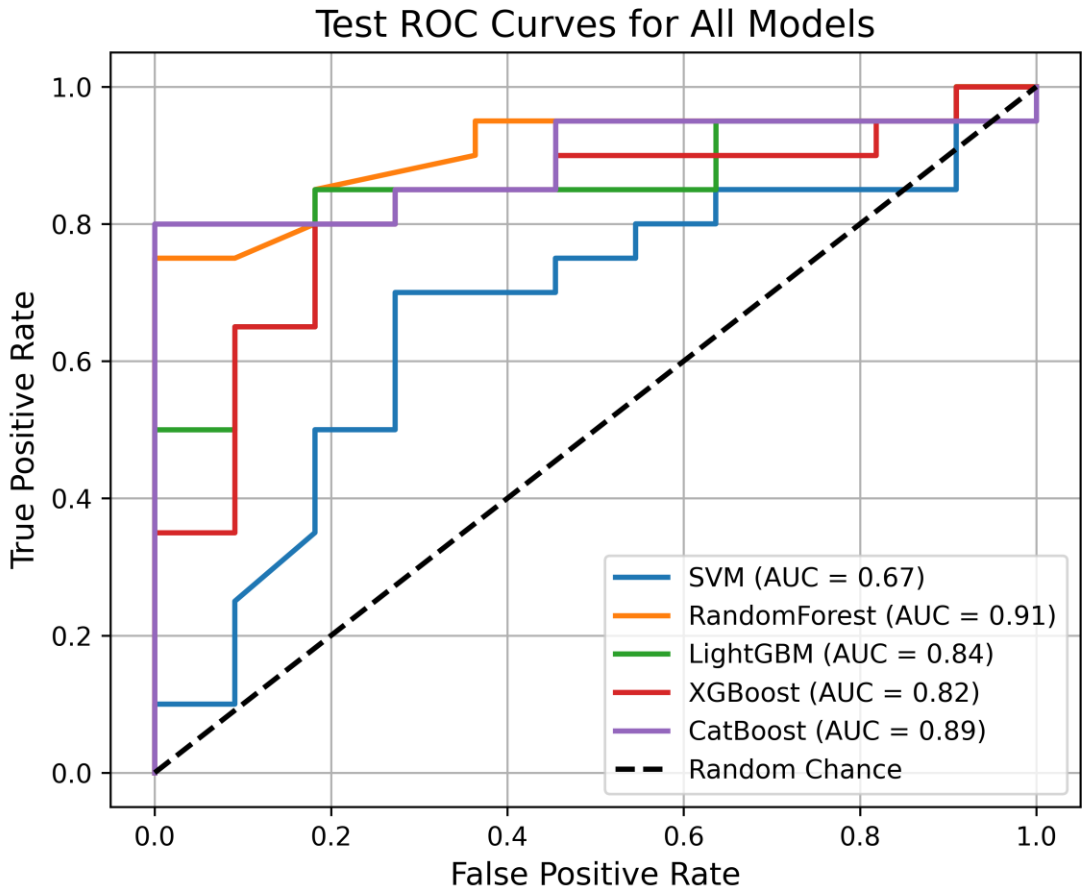

Supplemental Fig. 22 The performance of Clinical + NCCT + CBV + TTP Model.

Supplemental Table 22 Results of Clinical + NCCT+ CBV + TTP Model

Clinical + NCCT + CBV +TTP Model	Accuracy	Precision	Recall	Specificity	F1 Score	AUC
SVM	0.484	0.833	0.250	0.909	0.385	0.673
RandomForest	0.774	0.760	0.950	0.455	0.844	0.907
LightGBM	0.774	0.810	0.850	0.636	0.829	0.841
XGBoost	0.742	0.750	0.900	0.455	0.818	0.823
CatBoost	0.806	0.792	0.950	0.545	0.864	0.891
